# A Network‐Driven Framework for Drug Response Precision Prediction of Acute Myeloid Leukemia

**DOI:** 10.1002/advs.202506447

**Published:** 2025-07-11

**Authors:** Yinyin Wang, Rui Liu, Yinnan Zhang, Xiang Luo, Chengzhuang Yu, Shentong Fang, Ninghua Tan, Jing Tang

**Affiliations:** ^1^ Department of TCMs Pharmaceuticals School of Traditional Chinese Pharmacy China Pharmaceutical University Nanjing 211198 P. R. China; ^2^ Research Program in Systems Oncology Faculty of Medicine University of Helsinki Helsinki FI‐00014 Finland

**Keywords:** acute myeloid leukemia, drug sensitivity prediction, FLT3 Inhibitor, machine learning, network‐based analysis, precision medicine

## Abstract

Acute myeloid leukemia (AML) is a clonal malignancy of myeloid progenitor cells that demonstrates highly variable responses to current regimens, highlighting the need for precision medicine. However, reliable biomarkers for precision medicine treatment remain elusive due to cellular heterogeneity. Conventional Models based on bulk RNA sequencing and ex vivo assays often fail to capture the intricate molecular pathways and gene networks that underlie treatment response and resistance. Here, NetAML, a novel network‐based precision medicine platform that systematically develops 87 drug sensitivity prediction models for 87 clinical drugs using ex vivo drug responses from 520 AML patients with RNA‐Seq is presented. The approach leverages network‐based analysis and machine learning to identify biologically interpretable gene signatures that capture the complex molecular interactions driving differential drug responses. Notably, the signature genes derived from the models reveal distinct cellular mechanisms. For instance, the co‐expression of C19ORF59 and FLT3 is associated with resistance to FLT3 inhibitors. In summary, NetAML offers a powerful strategy for personalized AML treatment by constructing drug‐specific models, identifying clinically actionable biomarkers, and supporting the development of optimized, patient‐specific therapeutic regimens.

## Graphical abstract

A network‐based precision medicine platform, named NetAML is offered for personalized treatment of AML. It combines network analysis and machine learning to develop 87 models predicting drug sensitivity for clinical drugs. NetAML identifies interpretable gene signatures that drive differential drug responses, enabling the prediction of individual sensitivity while accounting for cellular heterogeneity. This facilitates personalized treatment strategies and biomarker discovery in AML.

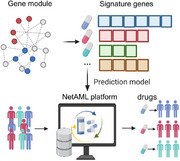

## 1 Introduction

Acute Myeloid Leukemia (AML) is a clinically and genetically heterogeneous disease, characterized by the clonal expansion of abnormal hematopoietic progenitors. Standard treatments for AML, such as induction chemotherapy and stem cell transplantation, yield a five‐year survival rate of only 29%.^[^ ^1^ ^]^ Despite advancements in chemotherapy, a significant proportion of AML patients are ineligible for intensive treatment due to age or comorbidities. Over the past five decades, targeted therapies—such as those aimed at FLT3^[^ ^2^ ^]^ and IDH1 mutations^[^ ^3^ ^]^ have broadened the therapeutic armamentarium in AML and yielded demonstrable improvements in patient survival. For instance, combining hypomethylating agents with Venetoclax has yielded superior overall survival compared to the conventional “3 + 7” regimen.^[^ ^4^ ^]^ Additionally, combination regimens incorporating gilteritinib with venetoclax and azacitidine have demonstrated encouraging efficacy, characterized by reduced toxicity profiles and higher complete remission rates.

However, these targeted therapies benefit only a subset of patients because the number of actionable targets is limited compared to the vast genetic diversity observed in AML.^[^ ^5^ ^]^ As a result, AML remains incurable, mainly, with most patients experiencing relapse and eventually developing multidrug resistance.^[^ ^6^, ^7^ ^]^ This underscores the urgent need to identify robust drug sensitivity biomarkers that can guide more effective and personalized treatment approaches.^[^ ^8^ ^]^ Recent advances in machine learning have enabled the integration of functional, molecular, and gene expression data to uncover complex molecular patterns underlying AML's genomic landscape and drug responses.^[^ ^9^ ^]^ For instance, studies employing non‐negative matrix factorization to integrate multi‐omics data have successfully revealed distinct network characteristics across AML subtypes, while other approaches using molecular descriptors and random forest models have pinpointed key properties associated with FLT3i bioactivity.^[^ ^10^, ^11^ ^]^ Despite these advances, most existing models tend to focus on single targets, thereby overlooking the complex biological processes and cellular heterogeneity that drive differential drug responses.

Cellular heterogeneity is a major contributor to variable drug responses in AML, as different subpopulations within a tumor may harbor distinct gene expression profiles and functional states. AML is increasingly recognized not as a single disease entity, but rather as a constellation of biologically and clinically distinct subtypes. These subtypes are defined by a spectrum of genetic, epigenetic, and microenvironmental alterations, resulting in heterogeneous disease trajectories and markedly variable responses to therapy. For instance, the bone marrow microenvironment differs significantly among patients, with interactions between leukemic stem cells and stromal components—mediated through cytokines and metabolic reprogramming—contributing to treatment resistance.^[^ ^12^ ^]^ Moreover, immune contexture disparities, such as T‐cell exhaustion and infiltration of immunosuppressive cells, further hinder the efficacy of emerging immunotherapies. At the molecular level, co‐occurring mutations and clonal evolution complicate the response to targeted therapies. Studies using ex vivo drug sensitivity assays have revealed that AML subpopulations at different disease stages or with distinct genetic backgrounds respond differently to the same therapeutic agents.^[^ ^13^ ^]^

However, conventional computational models for AML drug response prediction—such as non‐negative matrix factorization (NMF),^[^ ^14^ ^]^ regularized regression modelling (LASSO),^[^ ^15^ ^]^ and conditional inference forest methodology (cforest)^[^ ^16^ ^]^—often rely on oversimplified assumptions (e.g., linear gene‐gene interactions, static feature weights) and focus primarily on bulk gene expression data. These approaches struggle to distinguish response variations across subclones or cell types. As a result, conventional treatment regimens, which are primarily based on uniform protocols, often fail to account for the dynamic and multifactorial nature of AML resistance. Also, some generalized models (e.g., pan‐drug WGCNA clusters) ignore compound‐specific mechanisms. Models based on raw gene expressions or correlations often employ large feature sets or “black‐box” architectures, which limit mechanistic insights and clinical usability. These limitations underscore the urgent need for precision medicine approaches that integrate multidimensional data to tailor therapy to each patient's unique molecular and cellular context. In contrast, network‐based machine learning approaches offer the potential to capture the coordinated activity of functionally related gene sets within broader molecular regulatory networks. This not only deepens our understanding of pathogenic mechanisms but also enhances our ability to predict individual drug sensitivity, ultimately improving patient outcomes.^[^ ^18^ ^]^ Indeed, recent pharmacogenomic investigations have demonstrated the value of network models in identifying determinants of drug response.^[^ ^17^, ^18^, ^19^ ^]^

**Figure 1:**
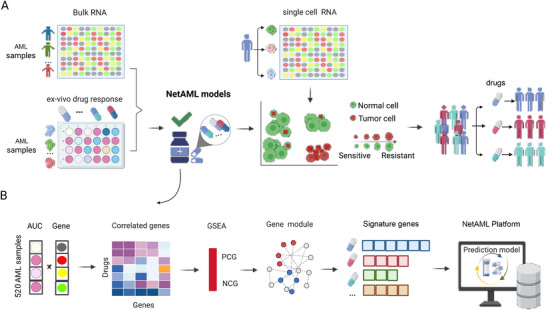
Overview of the NetAML platform. A) NetAML leverages RNA‐Seq data from 520 AML patients combined with ex vivo drug sensitivity data for 87 drugs to identify functional gene signatures associated with drug response. By accounting for cellular heterogeneity, NetAML provides individual predictions of drug sensitivity for clinical patients. B) By integrating a Disease Module Detection algorithm with 39 regression models, four types of features, and four feature selection methods, our approach builds drug‐specific predictive models that are both robust and biologically interpretable.

In this study, we introduce NetAML, a novel network‐based precision medicine platform specifically designed for AML (**Figure** **1**A,B). NetAML leverages RNA‐Seq data from 520 AML patients combined with ex vivo drug sensitivity data for 87 drugs to identify functional gene signatures associated with drug response. By integrating a Disease Module Detection algorithm with 39 regression models, four types of features, and four feature selection methods, our approach builds drug‐specific predictive models that are both robust and biologically interpretable. Crucially, the network‐based signature model captures the inherent cellular heterogeneity of AML, thereby illuminating the molecular basis for differential drug responses. Compared with existing methods, we have the following unique characteristics: 1) Drug‐specific PPI network modules: Unlike pan‐drug models, NetAML builds drug‐specific networks informed by functional annotations and known pathways, identifying fewer core genes per drug; 2) Model optimization across multiple configurations: We systematically evaluated multiple machine learning algorithms and feature selection strategies, selecting the best‐performing pipeline per drug; 3) Single‐cell validation of heterogeneity: We applied our gene signatures to scRNA‐seq data to resolve subclonal variation in drug response—an essential capability missing in prior models; 4) Interpretability and scalability: NetAML leverages transcriptomics alone, enabling cost‐effective, interpretable, and clinically scalable deployment. Together, NetAML addresses the limitations of prior models by integrating drug‐specific PPI networks, optimizing predictive pipelines for each compound, and accounting for cellular heterogeneity through bulk and single‐cell transcriptomic data. These innovations enable more accurate, interpretable, and mechanistically grounded predictions of drug responses, advancing the field toward precision AML therapeutics. In summary, this comprehensive framework not only facilitates the discovery of pharmacological targets and interacting biomarkers but also aligns with clinical outcomes, as demonstrated by the predictive accuracy of our model in an independent patient cohort. NetAML thus represents a novel strategy for personalized AML treatment.

## 2 Results

### 2.1 Ex Vivo Drug Response and Gene Expression of Samples

We developed NetAML, a network‐based machine learning platform integrating ex vivo drug sensitivity data and RNA‐seq data from 520 patients and 87 drugs from the OHSU study^[^ ^16^ ^]^ (Figure 1A,B).

**Figure 2:**
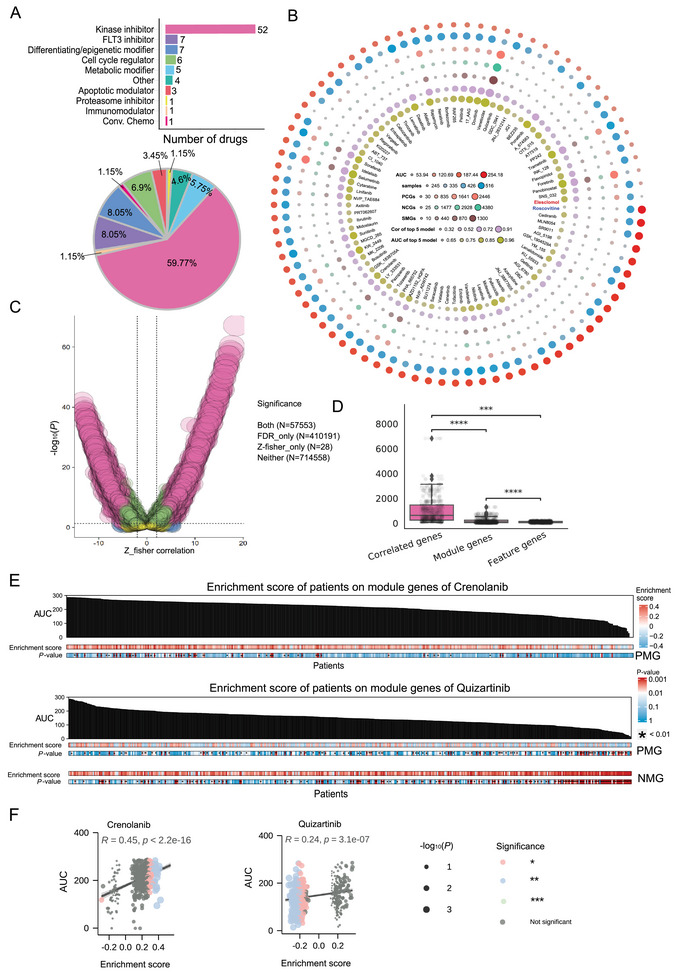
The minimum number of signature genes of drug response by network module and feature selection of machine learning methods. A) The distribution of drug classifications. B) The critical number of drugs, including their AUC, averaged AUC, relevant samples, PCGs from correlation analysis, NCGs from correlation analysis, CMGs from network module analysis, correlation of LR models, and drug response AUC of prediction models. C) Volcano plot of drug‐gene correlation with Z‐score from Fisher's z‐transformation and ‐log10(P) of the Spearman correlation analysis indicated by the false discovery rate (FDR). D) The boxplot shows the number of correlated genes that have decreased significantly from 526 SCGs to 121 module genes and further to 104 feature genes after the network module analysis and machine learning‐based feature selection (Mann‐Whitney test, ^*^ *P* < 0.05, ^***^ *P* < 0.001, ^****^ *P* < 0.0001). E) The enrichment analysis with PMGs and NMGs of Crenolanib and Quizartinib on AML patient samples to evaluate the discrimination ability of module genes for resistant and sensitive patients. A *P*‐value of less than 0.01 was marked with an asterisk, indicating significant enrichment of each patient's module genes. F) Pearson correlation analysis between the enrichment scores of module genes and drug AUCs.

We excluded low‐quality drugs and samples based on their drug response area under the curve (AUC) values to ensure the robustness of the data. Specifically, we observed that variance increased with the rise in AUC values (Figure S1A, Supporting Information), and the threshold for the top 5% AUC was found to be 277.33, based on the distribution of drug AUC. Therefore, the threshold of 277.33 was empirically derived as an extreme value from the observed distribution of AUC values across all samples and drugs (Figure S1B, Supporting Information). Here, we considered one drug with unreliable drug responses for model training and excluded it if 90% of the AUCs of this drug across 520 samples were larger than this extreme value. Therefore, Drugs with AUC values exceeding 277.33 in > 90% of samples were excluded, because such uniformity indicates minimal inter‐patient variability in response (i.e., the drug shows consistently poor efficacy across nearly all patients). Similarly, samples with AUC values exceeding 277.33 for all drugs were removed, as this uniformity likely reflects technical artifacts (e.g., measurement errors or systematic biases in viability assays), rather than true biological resistance. Additionally, drugs screened in fewer than 35 patients were excluded to maintain a reliable analysis.^[^ ^20^ ^]^ This resulted in 87 drugs being retained for further study, categorized as 52 kinase inhibitors (59.77%), 7 FLT3i (FLT3i) (8.05%), and seven differentiating/epigenetic modifiers (8.05%) (**Figure** **2**A). Despite their known efficacy, only seven FLT3i were identified, comprising 8.05% of the total 87 drugs for model training.

We excluded those with AUC values exceeding 277.33 for all drugs for sample selection, resulting in a final dataset of 520 samples. Gene expression data were obtained for 13,589 genes, selected based on raw count values exceeding 1 in at least 10% of the samples. The AUC values varied significantly across AML patients for different drugs (Figure 2B). Notably, Elesclomol exhibited the highest efficacy, with an average AUC of 53.94 across 447 samples, while Roscovitine showed the lowest efficacy, with an average AUC of 254.18 across 480 patient samples. Several FLT3i, such as JNJ_28312141, Quizartinib, and Dovitinib, also demonstrated relatively high sensitivity, with mean AUCs ranging from 141.97 to 150.97.

### 2.2 Identification of Drug‐Sensitivity‐Associated Genes

To better capture crucial genes associated with differential drug responses, we derived drug‐gene correlation pairs from 13,589 genes and 87 drugs. Drug‐gene correlations were converted to Z‐scores using Fisher's z‐transformation to normalize their distribution. To identify both strong and weak associated genes, we selected the top 5% of drug‐gene pairs based on their correlation: positively correlated genes (PCGs) with Z‐scores > 3.7 and negatively correlated genes (NCGs) with Z‐scores < −4.0 (Figure S1C, Supporting Information). These cutoffs were selected to capture the most extreme associations (both strong and weak) while striking a balance between statistical stringency and biological relevance. In detail, PCGs (Z‐score > 3.7) represent genes whose elevated expression correlates with reduced drug efficacy (e.g., resistance mechanisms). In contrast, NCGs (Z‐score < −4.0) correspond to genes whose low expression predicts enhanced drug sensitivity (e.g., sensitizing pathways). PCGs and NCGs were collectively termed sensitivity‐correlated genes (SCGs). Compared with standard Spearman correlation analysis of false discovery rate (FDR) between drugs and genes, our Fisher's z‐transformation identified 57,553 common drug‐gene pairs (Figure 2C). In contrast, our Fisher's z‐transformation identified 28 unique SCGs. The number of SCGs varied across drugs, ranging from 25 to 438 (Figure S1D,E, Supporting Information).

To explore drug similarities, we conducted hierarchical clustering of the SCGs for these 87 drugs (Figure S2A, Supporting Information). The drugs were clustered into six groups, with those within the same functional class showing substantially different SCGs, emphasizing the need for drug‐specific response signatures. Additionally, VEGFR inhibitors, such as Regorafenib and Cabozantinib, and PDGFR inhibitors, including Sunitinib, clustered with FLT3i, supporting their potential application in AML treatment. PKC inhibitors, such as LY333531, are observed to resemble FLT3 Inhibitors, including Sorafenib and Dovitinib, suggesting shared therapeutic properties. Interestingly, Bcl‐2 inhibitors (Bcl‐2i), Venetoclax and Elesclomol, formed a distinct group. Venetoclax, in particular, exhibited a remarkably high number of 2,446 PCGs and 4,380 NCGs, indicating its broad regulatory impact across a wide range of genes (Figure 2B). This finding underscores the unique characteristics of Venetoclax, which is frequently combined with FLT3i in AML treatment.^[^ ^21^ ^]^ Further analysis revealed that FLT3i (e.g., JNJ_28312141 and Quizartinib) and Bcl‐2i (e.g., Venetoclax and ABT‐737) exhibited distinct drug response profiles in AML patients, as reflected by low correlation coefficients (< 0.30). At the same time, FLT3i demonstrated high correlation among themselves (correlation > 0.70, Figure S2B,C, Supporting Information). Notably, these findings could not be revealed by clustering based on the AUC values between drugs and samples. The difference suggests the necessity of drug‐gene correlation analysis for individualized drug treatment by considering the gene expression patterns of patients. These results highlight the unique drug‐specific responses that stem from distinct gene expression phenotypes.

Then, we employed network‐based methods to extract functionally related genes involved in drug response. Gene Set Enrichment Analysis (GSEA) and HotNet community analysis^[^ ^22^ ^]^ were used to identify gene modules. After this step, one or two gene modules were determined for each drug, representing the most functionally correlated genes associated with drug sensitivity, which we here define as “module 1” and “module 2”. In detail, “module 1” is the largest and most important module, while “module 2” is the second important module. Following the network module analysis and machine learning‐based feature selection, the average number of correlated genes was significantly reduced from 526 SCG genes to 121 module genes and further to 104 feature genes (Figure 2D; Figure S3A, Supporting Information), highlighting the utility of these methods in identifying essential biomarkers for drug response.

We observed that some drugs may exert similar therapeutic effects by influencing the same pathways, despite differing SCGs. For example, when examining four FLT3i (Crenolanib, JNJ_28312141, Sorafenib, and Midostaurin) and Venetoclax, they shared eight upregulated and 37 downregulated pathways (Figure S3B, Supporting Information). Samples sensitive to Crenolanib were significantly enriched in its positively correlated module genes (PMGs), with a similar trend observed for Quizartinib (Figure 2E). Conversely, resistant samples showed enrichment in negatively correlated module genes (NMGs). The enrichment scores of PMGs were positively correlated with drug AUCs (Crenolanib: R = 0.45, *P* = 2.2e^−16^; Quizartinib: R = 0.24, *P* = 3.1e^−7^; Figure 2F), indicating that the identified module genes effectively reflect drug‐specific pharmacological responses. These findings demonstrate that integrating network analysis with machine learning yields interpretable, drug‐specific gene signatures.

### 2.3 Performance and Interpretation of Drug Response Prediction Models

**Figure 3:**
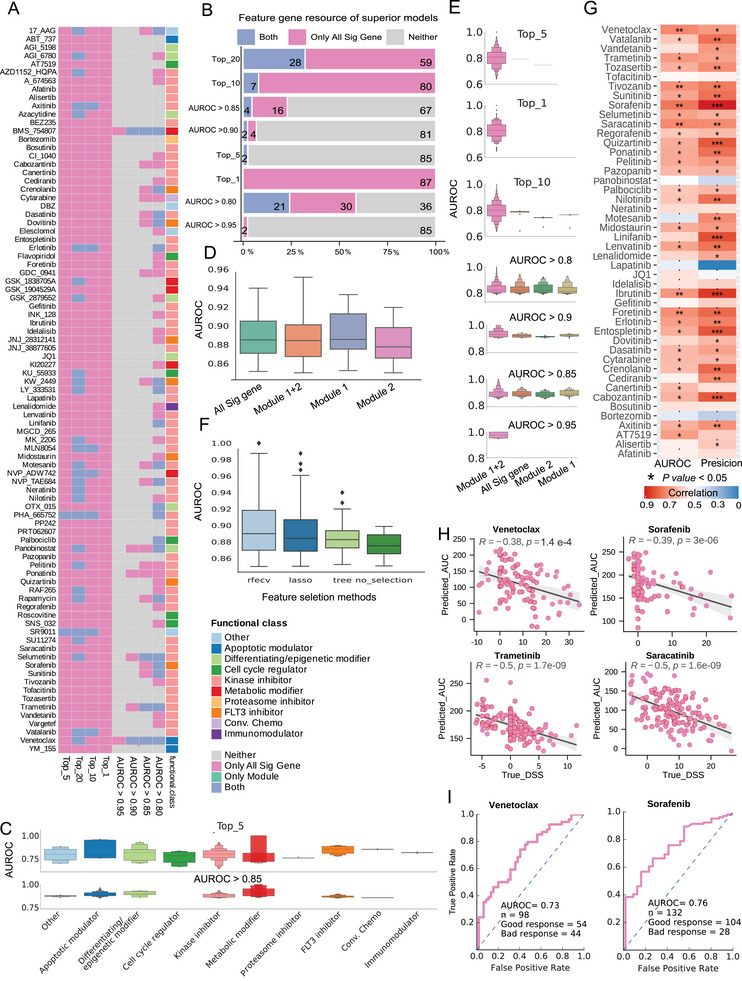
Performance of prediction models. A) The parameter distribution of the optimal models for the 87 drugs. B) Comparison of the top 1, 5, 10, and 20 models and the features of models with performance greater than 0.8, 0.85, 0.9, and 0.95 for the 87 drugs. C) The model's AUROC performance on drugs from different functional classes. D) Comparison of AUROC among different module types. E) Comparison of AUROC among different module types of the top 1, 5, 10, and 20 models separately. F) The AUROC performance of different feature selection methods. G) The external validation of the drug sensitivity prediction model of AUROC and precision from predicted AUC and DSS score using the FPMTB cohort, with ^*^ indicating the corresponding *value* > 0.50, ^**^ *value* > 0.60, ^***^ *value* > 0.70. H) The external evaluation of FLT3i models by Pearson correlation with predicted AUC as the Y axis and DSS as the X axis. I) The AUROC evaluation of FLT3i models with predicted AUC as the Y axis and DSS as the X axis.

To develop optimal prediction models, we performed 628 scenarios for each drug, incorporating 39 regression methods, four input feature types, and four feature selection methods with fivefold cross‐validation. The models were evaluated using the Area Under the Receiver Operating Characteristic curve (AUROC) for all 87 drugs (**Figure** **3**A). Notably, 51 of the 87 drugs achieved an AUROC greater than 0.8, with six drugs exceeding 0.90 (Figure 3B; Figure S4A, Supporting Information). Notably, when comparing the top 10 models per drug category, apoptotic modulators, conventional chemotherapies, and FLT3i exhibited significantly higher AUROC values than other drug classes (*P* < 0.05; Figure 3C; Figure S4B,C, Supporting Information). Among the top‐performing models, those trained with the second module as input features outperformed those using other feature types (Figure 3D,E; Figure S4A, Supporting Information). Additionally, models utilizing recursive feature elimination with cross‐validation (RFECV) outperformed those without feature selection (Figure 3F; Figure S4A, Supporting Information). After module analysis, twenty‐one drugs achieved excellent performance with AUROC values exceeding 0.80 (Figure 3B). Notably, the Bcl‐2i Venetoclax attained an AUROC of 0.93 with a correlation of 0.83 using 51 signature genes. In comparison, the FLT3i KW_2449 achieved an AUROC of 0.82 and a correlation of 0.66 with 67 signature genes, significantly reducing the number of necessary genes compared to models without feature selection.

These results validate the performance of our drug‐sensitivity models, which reliably forecast therapeutic response in AML cohorts. Furthermore, the network module analysis reveals functionally related gene sets that enhance the interpretability of these models, offering valuable insights into potential biomarkers for drug response.

### 2.4 External Validation of Prediction Models for Drug Sensitivity

To validate the predictive ability of the developed models, we utilized ex vivo drug sensitivity testing data from primary AML samples from the FPMTB study,^[^ ^20^ ^]^ which included 164 patient responses to 515 single agents.^[^ ^20^ ^]^ The prediction models were employed to estimate the AUC of drug responses for these patients, and we classified them into resistant and sensitive groups based on their mean Drug Sensitivity Scores (DSSs).

Among 47 common drugs, the predicted AUC values demonstrated intense discrimination, with 29 drugs achieving a precision greater than 0.60 (Figure 3G). When classifying patients into good and poor responders based on their mean Drug Sensitivity Score (DSS), 20 out of 47 drugs exhibited significant discrimination with *P* < 0.05. The Pearson correlation between predicted AUC and actual drug sensitivity was greater than 0.30 for 15 drugs, with a *P* < 0.05, yielding a mean correlation of 0.38 (Figure S5A, Supporting Information). Notably, for Bcl‐2i such as Venetoclax and FLT3i like Midostaurin, Quizartinib, and Sorafenib, Pearson correlations exceeded 0.35 (*P* < 0.05) (Figure 3H; Figure S5B, Supporting Information), with AUROC values greater than 0.70 (Figure 3I; Figure S5C, Supporting Information). These results confirm the ability of our signature‐based model to predict drug responses, supporting its clinical utility accurately.

### 2.5 Cellular Heterogeneity Drives Differential Drug Responses

Cellular heterogeneity plays a pivotal role in the varying drug responses observed in AML patients, as distinct cell populations exhibit different therapeutic responses.^[^ ^23^ ^]^ To identify cell types sensitive to FLT3 inhibition, we applied the EPIC^[^ ^24^ ^]^ method to infer the proportions of 11 leukemic cell types in pre‐therapy bulk RNA samples from the BeatAML cohort.

**Figure 4:**
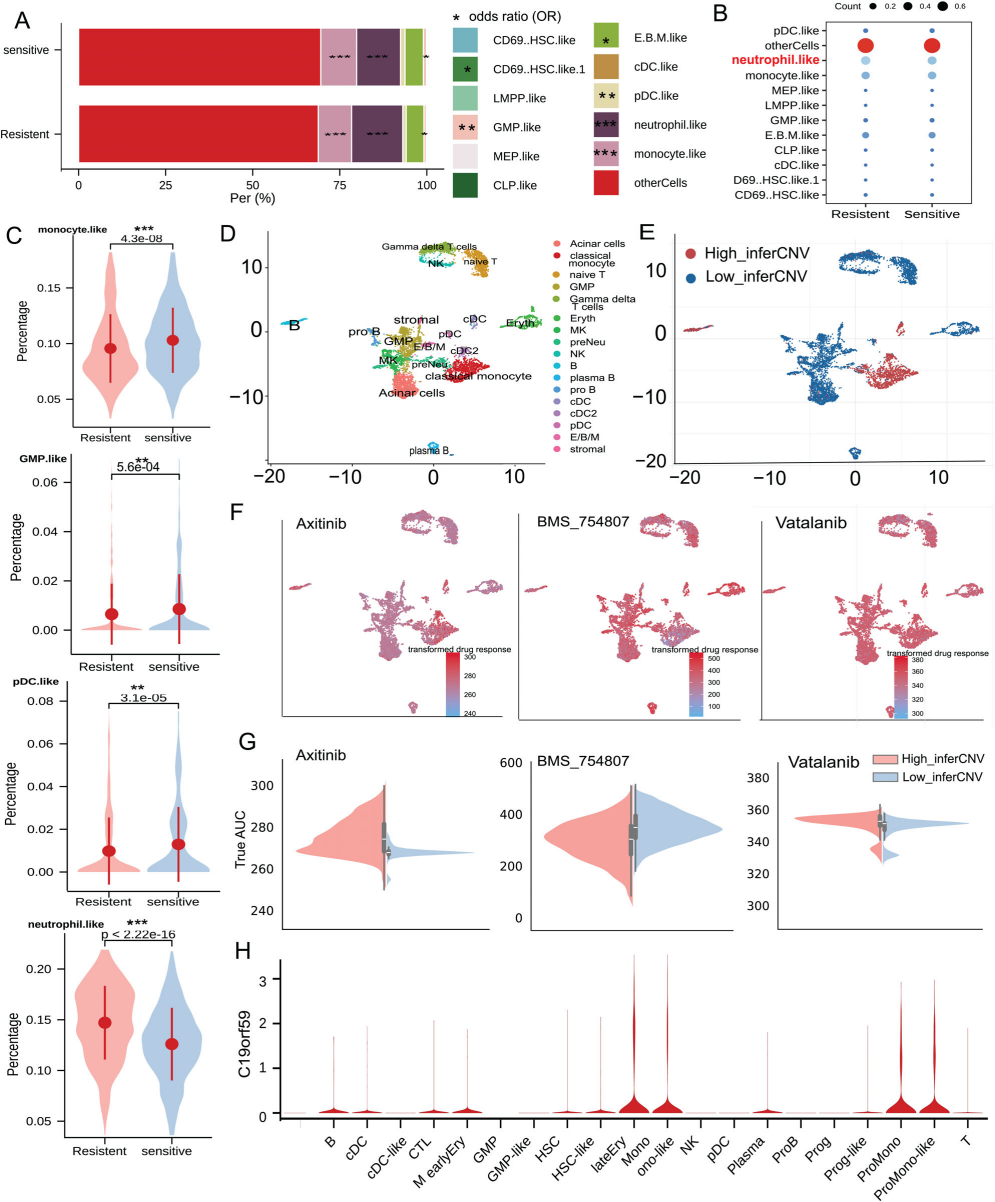
Cellular heterogeneity is associated with differential drug responses of AML patients. A) The average cell type percentage of sensitive and resistant patients separately. The odds ratio comparison of the cell type percentage among sensitive and resistant patients was inferred by the EPIC method. ^*^ *P* < 0.05, ^**^ *P* < 0.01, ^***^ *P* < 0.001. B) The average percentage of cell types from the EPIC method among sensitive and resistant patients. C) The comparison of cell type percentage from the EPIC method among sensitive and resistant patients using t‐test, ^*^ *P* < 0.05, ^**^ *P* < 0.01, ^***^ *P* < 0.001. D) 17 cell types were identified for AML patient P105 using the top 10 marker genes with the highest expression variation in each cluster. D) 17 cell types were identified for AML patient P105 using the top 10 marker genes with the highest expression variation in each cluster. E) Cells with a higher inferCNV score (High_inferCNV) of AML patient P105 were distinguished from cells with a lower inferCNV score (Low_inferCNV). F) The predicted drug response value for AML patient P105 based on single‐cell RNA sequencing data is used to prioritize optimal drugs. G) Drug sensitivity of AML patient P105 varied among High_inferCNV and Low_inferCNV cells for different drugs. H) Comparison of FLT3i signature genes C19orf59 across different Cell subtype populations.

We classified 92 patient samples into Resistant and Sensitive groups based on the median ex vivo drug response AUC to FLT3i. EPIC utilized gene expression features to infer the fraction of each cell type for each sample based on single‐cell data of AML by van Galen et al.^[^ ^25^ ^]^ First, the average abundances of cell types among sensitive and resistant patients are presented in **Figure** **4**A. Then, the Odds ratio (OR) of cell type percentage among sensitive and resistant patients is inferred by the EPIC method by assigning patients to the cell type with maximum possibility. Our analysis revealed that neutrophil‐like cells were significantly more abundant in the Resistant group (14.71%) compared to the Sensitive group (12.60%) (Figure 4B). In contrast, monocyte‐like cells were more abundant in the Sensitive group (10.29%) compared to the Resistant group (9.56%), suggesting that certain cell types are associated with drug sensitivity and resistance. Compared to the possibility of resistant and sensitive patients, we also observed a higher abundance of plasmacytoid dendritic cell‐like (pDC‐like) and granulocyte‐monocyte progenitor‐like (GMP‐like) cells in the Sensitive patients compared to the Resistant group, with P‐values of 3.10e^−5^ and 5.60e^−4^, respectively (Figure 4C; Figure S6, Supporting Information, T test), which aligns with previous findings at the single‐cell level.^[^ ^26^ ^]^ Further analysis of the distribution of signature genes across cell subpopulations showed that genes such as C19ORF59, IL2RA, RHOU, BIK, and CEACAM8 were highly expressed in plasmacytoid dendritic cell‐like (pDC‐like) and granulocyte‐monocyte progenitor‐like (GMP‐like) cells (Figure S7, Supporting Information). This aligns with our AUC distribution across different cell types, further illustrating the role of cellular heterogeneity in drug response. Notably, we noticed the high proportion of ‘other cells’ (≈70%) unannotated leukemic populations. This might be caused by AML's inherent heterogeneity, where malignant cells often lack canonical immune markers. Therefore, we performed additional deconvolution using CIBERSORTx^[^ ^27^ ^]^ (a method optimized for hematological malignancies). We also found that the FLT3i‐resistant group exhibited significantly enriched naive B cells, monocytes, M0 macrophages (poised for M2 polarization), and Tregs (*P* < 1e^−5^). In contrast, the FLT3i‐sensitive group displayed a pro‐inflammatory milieu dominated by resting CD4+ memory T cells, mast cells, neutrophils, and resting NK cells (*P* < 1e^−5^, Figure S6B–D, Supporting Information). These results further confirmed our findings with EPIC.

To validate the predictive capability of our models, we applied them to AML patient samples, prioritizing optimal drugs based on single‐cell RNA sequencing (scRNA‐seq) data. We first downloaded the scRNA‐seq FASTQ files for the AML patient (ID: P105) from the Genome Sequence Archive for Human at the BIG Data Center of the Beijing Institute of Genomics, Chinese Academy of Sciences.^[^ ^28^ ^]^ For cell type annotation, 17 cell types were identified using the top 10 marker genes with the highest expression variation in each cluster (Figure 4D). Malignant cells were distinguished from normal cells using inferCNV. This tool analyzes copy number variation (CNV) patterns to identify malignant cells (Figure 4E). The inferCNV method could identify malignant cells based on large‐scale genomic aberrations. The highest inferCNV scores were observed specifically within classical monocyte and B cell clusters (Figure 4D,E), which is biologically consistent with AML pathology. AML blasts frequently adopt monocytic (M4/M5 subtypes) or, less commonly, B‐lymphoid phenotypes, as malignant cells mimicking these immune subtypes would exhibit non‐diploid CNV profiles, explaining their high inferCNV scores. This aligns with established literature where leukemic blasts display monocytic differentiation as a disease‐defining feature, often enriched at relapse.^[^ ^29^ ^]^ Notably, the inferCNV score is consistent with our previous cell deconvolution analysis, which shows the monocyte population was significantly enriched in FLT3i‐resistant samples (Figure 4A–C). This mirrors clinical observations linking monocytic differentiation and aberrant monocyte expansion to chemoresistance, relapses, and leukemic stem cells.^[^ ^30^, ^31^ ^]^

We then applied a larger scale of 87 drug models to predict drug responses for each cell in the scRNA‐seq dataset. The area under the curve (AUC) was used as the evaluation metric, with lower AUC values indicating stronger drug efficacy. AUC values were transformed for comparative analysis, and drug response profiles for each cell were visualized. With drug sensitivity model panels to predict the drug sensitivity of individual cells, NetAML would facilitate the optimal selection of drugs for individuals based solely on their pharmacogenomic features. For instance, Axitinib showed higher sensitivity to high‐CNV cells than low‐CNV cells in patients P105 (Figure 4F,G). Conversely, BMS_754807 exhibited higher sensitivity in low‐CNV cells, suggesting potential off‐target effects. Vatalanib showed resistance across both groups. This differential drug response strongly supports the biological distinction captured by the inferCNV classification.

### 2.6 A Pan‐Drug Gene Signature Panel for AML to Reveal Functional Biomarkers and Therapeutic Targets

**Figure 5:**
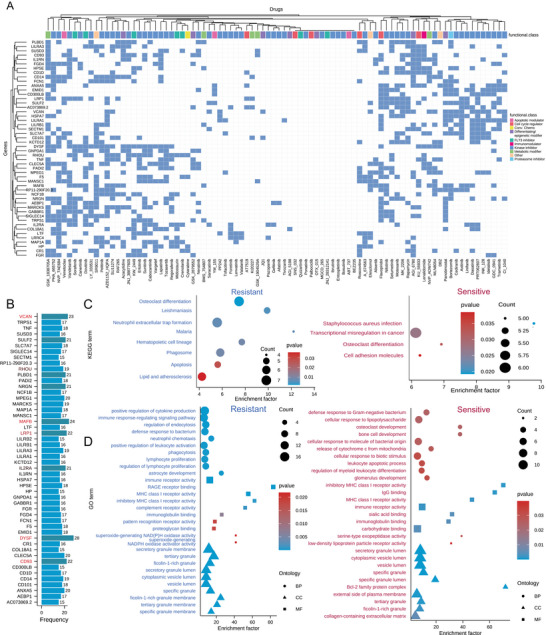
Landscape panel of drug signatures for AML. A) The heatmap of gene signatures between 87 drugs and the top 50 most frequent genes. B) The frequency of top signature genes. C) Comparison of ontology functions of gene signatures from resistant and sensitive drugs. D) Comparison of pathways using gene signatures from resistant and sensitive drugs.

Gene signatures identified in our models underpin precision drug sensitivity in AML. A comprehensive pan‐drug analysis of our top five models for 87 AML drugs identified a landscape of 3,407 signature genes (**Figure** **5**A). Among these, the most frequently identified genes were DYSF (detected in 28 drugs), MAFB (24), VCAN (23), CD93 (22), and LRP1 (22) (Figure 5B), some of which had been reported in previous studies.^[^ ^32^, ^33^ ^]^ DYSF (Dysferlin) is a calcium‐sensitive protein critical for membrane repair and vesicle trafficking in cells. In AML, dysregulated expression of DYSF may compromise leukemic cell membrane integrity, potentially altering stress adaptation mechanisms. Recent studies suggest impaired membrane repair could enhance susceptibility to chemotherapy‐induced damage, while overexpression might promote survival by mitigating drug‐triggered membrane destabilization. Additionally, dysferlin's role in intracellular signaling (e.g., calcium flux modulation) may indirectly influence apoptosis resistance, a hallmark of AML persistence,^[^ ^34^ ^]^ and could be used as a prognostic biomarker to predict survival in patients with renal cell carcinoma.^[^ ^35^ ^]^ Meanwhile, MAFB is a transcription factor essential for myeloid differentiation, particularly in monocyte/macrophage lineage commitment. In silico stratification analyses of molecular data from AML patients revealed a reciprocal relationship between MYB and MAFB expression, highlighting a novel biological interconnection between these two factors in AML and supporting new rationales for targeting MAFB in MLL‐rearranged leukemia.^[^ ^36^ ^]^ MAFB has been reported to promote cancer stemness and tumorigenesis in osteosarcoma through a Sox9‐mediated positive feedback loop.^[^ ^37^ ^]^ These results suggest that our model can effectively capture drug response biomarkers for AML. Notably, the top 50 signature genes were linked to 403 drug‐gene pairs across 24 drugs, underscoring the crucial role of these top genes. This result also suggests that our network‐based analysis can capture the general AML treatment‐related biomarkers.^[^ ^38^ ^]^

Intriguingly, our analysis revealed that the Bcl‐2i Venetoclax and PKC inhibitor LY_333531 share common signature genes with FLT3i (e.g., Sorafenib and Dovitinib), suggesting potential combination strategies (Figure 5A; Figure S2A–C, Supporting Information). In contrast, Quizartinib, JNJ_28312141, and gilteritinib overlap significantly with ABT‐737, a Bcl‐2 inhibitor. Moreover, comparing sensitive versus resistant AML drugs showed that osteoclast differentiation is a key process (Figure 5C) and that signature genes from sensitive drugs are enriched in functions related to transcriptional misregulation and cell adhesion—processes critical for maintaining hematopoietic stem cell (HSC) quiescence and proliferation. GO analysis further revealed enrichment of Bcl‐2 family protein complex functions among these genes (Figure 5D), supporting the rationale for combining Bcl‐2i with FLT3i and chemotherapy.

Community analysis of the drug–gene network and protein–protein interaction (PPI) network further identified 41 gene pairs in signatures of more than 10 drugs (Figure S8A, Supporting Information). Here, DYSF and SULF2 emerged as hub genes, with FLT3 and DYSF consistently appearing in 11 drugs. Clustering identified three primary gene modules (Figure S9, Supporting Information) and four functionally connected modules within the network (Figure S8B, Supporting Information). Notably, the shared signatures between the HDAC inhibitor panobinostat and Venetoclax (Figure S8C, Supporting Information) hint at similar gene regulation patterns, reinforcing the potential of combination therapies for patients who have failed prior treatments.

### 2.7 Clinical Predictive Value of FLT3i Gene Signatures

Given the clinical importance of FLT3 mutations in AML, we characterized a gene signature landscape specific to FLT3 inhibitors (FLT3is), comprising 629 genes. Among these, only 47 genes appeared as signatures in at least three FLT3is. The six most frequently co‐expressed genes—IL2RA (*n* = 5), RHOU (*n* = 4), C19ORF59 (*n* = 4), BIK (*n* = 4), C10orf128 (*n* = 4), CEACAM8 (*n* = 4), and OAT (*n* = 4)—were identified (Figure 5A; Figure S9A, Supporting Information). Besides common pathways such as osteoclast differentiation, these genes are also linked to the B cell receptor signaling pathway (Figure S9B, Supporting Information), underscoring the potential role of B cells in mediating sensitivity to FLT3i. Community analysis of FLT3i signatures revealed that the top six genes form tightly interconnected modules (Figure S9C, Supporting Information). Although FLT3 itself is not frequently identified, it emerged as a hub within the co‐expression network (Figure S10, Supporting Information), closely connected to IL2RA and BIK, while SULF2 was also a critical hub.^[^ ^39^ ^]^

**Figure 6:**
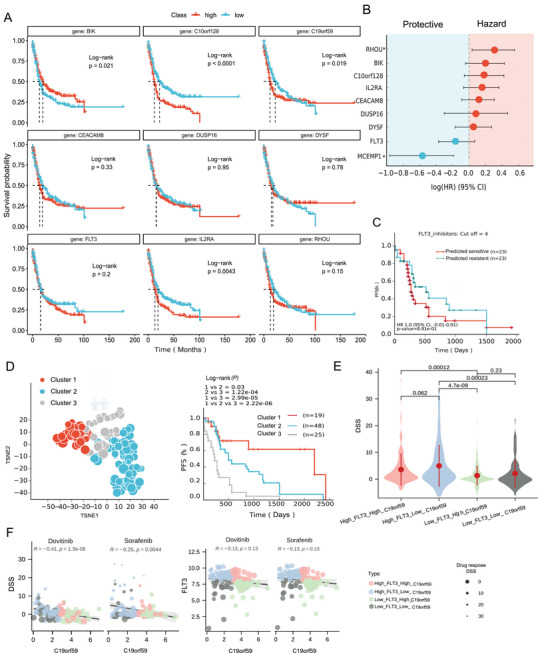
Clinical validation of FLT3‐i signature gene with varied survival time of FPMTB cohort. A) The Kaplan–Meier curve of AML patients grouped by individual signature genes of the nine top signatures from FLT3i. B) The Cox hazard value of individual signature genes in AML patients. C) The Kaplan–Meier curve discrimination of AML patients grouped by predicted AUC on FPMTB patients with FLT3 mutation. D) The TSNE plot of FPMTB AML patients clustered into three groups based on expression of ten FLT3 signature genes, as well as their Kaplan–Meier curve comparison by log‐rank test. E) The comparison of drug sensitivity DSS between different types of drugs by grouping FPMTB patients by FLT3 and C19ORF59 genes using a t‐test. F) The Pearson correlation between the expression of C19ORF59 and FLT3‐i drugs/FLT3 gene of FPMTB patients, with color indicating different groups of genes and size representing the drug response.

To evaluate the predictive utility of FLT3i gene signatures, we analyzed 92 patients with AML from the FPMTB cohort who harbored FLT3 mutations. Patients were stratified based on the expression of seven frequently identified FLT3i signature genes (including FLT3 and DYSF) into four groups (high, higher, lower, and low expression). Log‐rank tests revealed significant differences in survival (*P* < 0.05; **Figure** **6**A). Notably, high expression levels of BIK were associated with favorable outcomes, whereas the expression of IL2RA and C19ORF59 correlated negatively with survival. This result is consistent with previous observations that overexpression of IL2RA, for example, has been associated with chemotherapy resistance and poor prognosis in AML.^[^ ^38^ ^]^ Hazard ratio analysis further identified C19ORF59 (MCEMP1) as a significant risk factor (HR = 0.50, 95% CI = 0.32–0.79, *P* = 0.0038; Figure 6B). Furthermore, by integrating the predicted AUC values from our models for seven FLT3is, patients were classified as pre‐sensitive (top quartile) or pre‐resistant (bottom quartile). The pre‐sensitive group exhibited significantly longer survival time (Figure 6C). Clustering analyses based on the top‐frequency signature genes further delineated distinct patient groups with different survival times (Figure 6D), a pattern also reflected in t‐SNE visualizations. These findings demonstrate that FLT3i‐specific gene signatures serve as robust predictors of clinical response in AML.

To further explore the biological relevance of these hub genes, we examined the relationship between these signature genes and mutational status in 520 patients from the BeatAML cohort, using Crenolanib as a case study. Heatmap analysis revealed that patients were clustered into three groups with distinct drug response AUCs (sensitive, intermediate, and resistant; Figure S11A, Supporting Information), indicating that the signature genes can effectively discriminate patients. Patients with FLT3 and NPM1 mutations generally exhibited greater sensitivity to FLT3is, whereas those with TP53 mutations were more resistant. IL2RA expression showed a negative correlation with drug response AUC. Moreover, network proximity analysis using FLT3i Quizartinib demonstrated that the signature genes and target genes are significantly closer than random sets (network distance = 2.58, Z‐score = −1.76, *P* < 0.07, Figure S11B, Supporting Information). For instance, the gene MAPK15 (Q8TD08) directly interacts with the signature gene DUSP1, and OAT interacts with multiple FLT3 targets (e.g., ABL1, CBL, KIT), indicating that these module genes, derived from protein‐protein interaction networks, effectively represent the pharmacological landscape of drug‐specific responses.

### 2.8 C19ORF59 as a Novel Determinant of FLT3i Resistance

FLT3 expression and mutation are established indicators for FLT3‐targeted therapies. In this study, we identified C19ORF59 as an essential biomarker gene. C19ORF59 has been reported as a potential therapeutic biomarker associated with immune infiltration in the advanced gastric cancer microenvironment.^[^ ^40^ ^]^ In the FPMTB cohort, our analysis also reveals that C19ORF59 plays a pivotal role in modulating FLT3i sensitivity (Figure S12, Supporting Information). In our previous study, C19ORF59 also exhibits high expression in monocytic lineage populations, including monocytes (Mono), monocyte‐like precursors (Mono‐like), promonocytes (ProMono), and promonocyte‐like cells (ProMono‐like) (Figure S4H, Supporting Information), suggesting its potential association with monocytic lineage expansion and leukemogenesis. However, its biological role in the drug resistance of AML for FLT3i remains underexplored.

Therefore, we stratified patients from the FPMTB cohort into four groups based on the co‐expression of C19ORF59 and FLT3 (C19ORF59⁺FLT3⁺, C19ORF59⁺FLT3^−^, C19ORF59^−^FLT3⁺, and C19ORF59^−^FLT3^−^). Our analysis demonstrated that among patients with high FLT3 expression, those with lower C19ORF59 levels (C19ORF59⁻FLT3⁺) exhibited significantly improved drug sensitivity compared to their counterparts (Figure 6E; Figure S13, Supporting Information). Correlation analysis revealed a negative association between C19ORF59 expression and drug sensitivity for four of five FLT3i (correlation coefficients ranging from −0.24 to −0.41, *P* < 0.01; Figure 6F; Figure S14A, Supporting Information). These data suggest that the modulation of C19ORF59 could be a strategic target to overcome FLT3i resistance. The negative correlation between FLT3 and C19ORF59 further supports a compensatory mechanism that may be exploited to improve patient outcomes (Figure S14B, Supporting Information).

Our integrated network‐based analysis has uncovered robust gene signatures that characterize the precision drug sensitivity of AML across a wide range of therapies, providing clinically relevant predictors of response to FLT3 Inhibitors. Importantly, identifying C19ORF59 as a novel modulator of FLT3i resistance highlights its potential as a therapeutic target for overcoming drug resistance in AML. These findings underscore the translational potential of our AI‐derived gene signatures in guiding personalized treatment strategies for AML.

## 3 Discussion

In this study, we developed NetAML, a novel network‐based precision medicine platform for AML that integrates network analysis with machine learning‐based feature selection to identify gene signatures associated with drug responses. By constructing separate predictive models for 87 drugs, each characterized by unique module genes and enriched pathways, we have established a drug‐sensitive signature landscape that not only elucidates the mechanisms of drug resistance but also provides a powerful tool for pre‐diagnosing clinical responses in AML patients.

NetAML represents a paradigm shift in precision medicine for AML. Traditional approaches in drug design often adhere to a “one gene, one drug, one disease” model, which oversimplifies the complex, multifactorial nature of cancer. In contrast, our platform acknowledges that the interplay of multiple genes drives AML and that compensatory mechanisms often mask the effects of targeting a single gene. By leveraging network‐based methods, NetAML systematically identifies functionally related gene modules within the protein‐protein interaction (PPI) network, providing a more comprehensive understanding of the biological processes underlying drug responses. In this study, we selected HotNet^[^ ^22^ ^]^ as the core algorithm for module detection due to its heat diffusion–based network propagation framework, which effectively captures both direct and indirect gene‐gene interactions within protein–protein interaction (PPI) networks. Compared to other network‐based methods such as dne,^[^ ^41^ ^]^ jActiveModules^[^ ^42^ ^]^ and Net‐Cox,^[^ ^43^ ^]^ HotNet demonstrates more stable module detection across heterogeneous datasets. This robustness makes it particularly well‐suited for analyzing high‐dimensional and noisy transcriptomic data. Moreover, HotNet has been shown to identify functionally coherent modules relevant to complex disease mechanisms, enhancing its biological interpretability. Importantly, HotNet operates in an unsupervised manner, aligning well with the aims of our study, which focuses on uncovering drug response–associated gene modules using only transcriptomic profiles and network topology, without relying on phenotype labels or survival outcomes. This characteristic broadens its applicability to diverse biomedical contexts where such annotation data may be limited or unavailable.

Our results demonstrate that NetAML can capture the most impactful genes through rigorous network module analysis, thereby reducing the number of required signature genes while maintaining high prediction accuracy. This streamlined approach enhances our ability to predict drug response and paves the way for discovering novel biomarkers that could guide the selection of personalized treatment strategies. For instance, patients exhibiting poor reactions to the FLT3i Crenolanib were found to harbor both TP53 and FLT3 mutations. Notably, cells with TP53 knockout showed significantly decreased sensitivity to Crenolanib, underscoring the need for treatment strategies targeting both mutations in such patients.^[^ ^44^ ^]^ For example, when compared to the model from Tyner et al.,^[^ ^15^ ^]^ which employed single‐model LASSO regression across all drugs, NetAML systematically evaluated 39 machine learning algorithms (including SVM, random forest, and gradient boosting) with four feature selection strategies, selecting the optimal pipelines for each drug. Our drug‐specific model achieved superior predictive accuracy (mean correlation 0.61 across 87 drugs). In addition, NetAML generated concise signatures (≈110 genes/drug on average) compared to the pan‐drug model's larger feature sets, thereby enhancing biological translatability. Another recent model is based on Pino et al.'s framework,^[^ ^14^ ^]^ which requires proteomic/phosphoproteomic data. However, expensive and low‐throughput essays may limit clinical adoption. Notably, NetAML in this study achieved comparable or better performance using transcriptomics alone (mean correlation 0.61 vs Pino et al.'s reported mean correlation 0.30) (Figure S15, Supporting Information). For example, for drugs like Venetoclax and Panobinostat, Pino et al.'s linear models required 905 and 978 protein or phosphosite features, respectively. In contrast, NetAML's network‐guided model achieved similar accuracy with ≈230 and 200 genes, respectively. More importantly, unlike models trained solely on cell lines (e.g., DrugCell^[^ ^45^ ^]^), NetAML uses patient‐derived data, offering greater potential clinical relevance despite potentially high in silico performance of cell line‐based models. In the future, such an advanced framework could also be employed, along with the accumulation of patient‐derived data, to enhance clinical translation.

One of the significant challenges in AML treatment is cellular heterogeneity, which contributes to differential drug responses among patients. AML tumors comprise diverse cell subpopulations, each with distinct gene expression profiles and functional characteristics. Traditional bulk analysis methods often overlook these nuances, limiting the accuracy of drug response predictions. Our network‐based signature model, however, is uniquely positioned to account for this heterogeneity. By identifying gene modules that reflect critical biological processes, such as osteoclast differentiation, cell adhesion, and stemness, our model captures the complex interactions within tumor cell subpopulations. This approach provides deeper insights into why specific cell subsets exhibit resistance to therapy while others remain sensitive. For example, gene signatures associated with the Bcl‐2 family and cell adhesion molecules have been shown to play pivotal roles in modulating drug response. By incorporating these multifaceted interactions, NetAML enhances predictive accuracy and offers a robust framework for understanding the molecular underpinnings of differential drug responses driven by cellular heterogeneity.

Our study links ex vivo drug responses to in vivo clinical outcomes by analyzing drug screening data from 520 AML patients across 87 different drugs. Integrating network‐based gene signatures with clinical data provides a more straightforward interpretation of expression profiles concerning patient survival and treatment efficacy. This is particularly important given that many conventional models based solely on cell line data fail to capture the complexity of human disease. Despite these advances, our model has certain limitations. It primarily relies on transcriptomic data, while other genomic factors such as mutations and copy number variations also play significant roles in drug response. Additionally, validating our model in a clinical context remains challenging due to the common practice of multi‐drug treatments in AML. Future studies integrating multi‐omics data and longitudinal clinical outcomes will be crucial for further refining and validating the NetAML platform.

In summary, NetAML provides a novel and powerful approach to personalized AML treatment by combining network‐based analysis with machine learning. Our strategy identifies robust gene signatures that serve as biomarkers for drug response and effectively captures cellular heterogeneity, a key driver of differential drug responses in AML. These findings underscore the potential of NetAML to transform precision medicine in AML by guiding personalized treatment strategies and facilitating the discovery of new therapeutic targets.

## 4 Experimental Section

### Patient Materials and Data Pre‐Processing

The BeatAML dataset was collected from Oregon Health & Science University (OHSU).^[^ ^16^ ^]^ Compiled over a decade, this dataset comprised samples from AML patients, featuring in vitro drug sensitivity testing, clinical annotations, and DNA and RNA sequencing. It aimed to enhance the understanding of the factors influencing drug response and clinical outcomes in AML. The dataset comprised a cumulative cohort of 942 specimens from 805 patients, including the initially published “Wave 1 + 2” cohort, which consisted of 649 specimens from 526 patients, and the recently published “Wave 3 + 4” cohort, containing 293 specimens from 279 patients. These samples represented various AML conditions, including newly diagnosed, transformed, and treatment‐related cases, with 70% classified as freshly diagnosed and a smaller proportion as relapsed or residual disease cases.

In this study, only the first available specimen for each patient within a five‐day interval was utilized, ensuring that a single sample for each data type represents each patient. Samples collected during remission were excluded. The dataset was manually curated from patient electronic health records, encompassing clinical, prognostic, genetic, cytogenetic, and pathological laboratory values, as well as treatment and outcome data. Additionally, it included multi‐dimensional data on gene characteristics of various leukemia samples, such as common hematologic malignancy mutations, DNA and RNA sequencing, FLT3‐ITD detection, and four‐base pair NPM1 insertion detection, as well as in vitro drug sensitivity testing.

The in vitro drug sensitivity testing assessed the response to 166 common drugs across 542 samples, measuring the area under the curve (AUC) for drug sensitivity. Two screening criteria for this study were applied: 1) the in vitro response data must include at least 35 samples, and 2) RNA‐seq data must be available. Ultimately, 87 drugs from the BeatAML dataset were retained. To explore the characteristics of different drug responses, these drugs were categorized into ten classes based on drug type and target activity, referencing information from MedChemExpress, Selleck, and Cell Signaling Technology.

### Network Module Analysis for Capturing Drug Response‐Related Genes

The correlation coefficients were calculated between gene expression and drug response data to identify genes associated with each drug. From the samples of AML patients, positive and negative drug‐gene correlations for 87 drugs was derived. The top 5% of drug‐gene correlations (Z‐scores) were deemed significant. Each drug was thus associated with a unique set of significant positive and negative genes, referred to as sensitivity‐correlated genes (SCGs), which play roles in drug response mechanisms.

To evaluate the pharmacological effects of these SCG genes, a gene set enrichment analysis (GSEA) for each drug was conducted. Unlike the conventional approach, which ranks genes by expression values, SCG genes were ranked by correlation coefficients. SCGs present in any enriched pathways with a *P* < 0.05 was retained.

Additionally, the HotNet algorithm was employed to identify gene modules that exhibit close interactions within the PPI network. To identify drug‐specific gene modules within the protein–protein interaction (PPI) network, HotNet2, which detects groups of genes that are both functionally related and topologically proximal was employed. The diffusion threshold was set to 0.5, controlling the extent of heat propagation across the network—higher values favor tightly connected modules. In comparison, lower values allow the inclusion of broader, potentially indirect associations. 1,000 permutations were performed to assess the statistical significance of identified modules. The baseline heat was set to 0, so that non‐scored nodes (genes not in the initial seed list) would not appear in the resulting clusters. HotNet2 was iteratively ran, removing genes from the largest identified module each time and rerunning the analysis, until no modules with more than five genes remained or statistical significance was not met (*P* > 0.05). A minimum module size of 5 genes was enforced, with a maximum of 10 iterations per run. A consensus cutoff of 2 to enhance robustness, retaining only modules detected in at least two iterations was applied. The PPI networks in the study were constructed using the STRING database^[^ ^46^ ^]^ (https://string‐db.org/), which included interactions with confidence scores greater than 700 as high‐confidence links, following the methodology established by Fernández–Torras et al.^[^ ^18^ ^]^ Specifically, the SCGs were ranked by the drug‐gene correlation coefficients for each drug and then mapped to the PPI network. For module expansion, the Disease Module Detection (DIAMOnD^[^ ^47^ ^]^) algorithm was utilized, which identifies genes closely interacting with seed nodes in the PPI network that were not initially classified as drug‐associated. Seed genes for each drug were selected based on drug‐gene correlation coefficients derived from transcriptomic data, with a focus on SCGs. The algorithm iteratively added genes up to 200 nodes, a cutoff proposed in the original DIAMOnD study based on orthogonal functional validation. A seed weight (alpha) of 10 was assigned to prioritize initial seed genes during module expansion. Confidence scores from the STRING database were incorporated as edge weights by default, favoring biologically relevant interactions. As a result, a panel of drug‐specific gene modules closely related to drug responses was obtained, which would serve as features for subsequent machine‐learning models. Positive and negative module genes were treated as separate gene sets to compute enrichment scores.

### Developing Optimal Regression Models to Predict Drug Response

39 different linear regression machine learning methods were employed to predict the AUC of drug response based on the gene expression profiles of AML patients. Network‐based analyses often generate hundreds of feature genes, which could potentially affect the generalization performance of machine learning models and lead to overfitting. Therefore, three standard feature selection methods were utilized to identify the most contributive features and avoid overfitting: Recursive Feature Elimination (RFE), Lasso, and tree‐based estimators. The hyperparameter selection strategy for the 39 regression models is listed in Table S1 (Supporting Information).

To obtain optimal models and hyperparameters for predicting drug sensitivity, SCGs and module genes as feature datasets were used. Each drug could be associated with one or two gene modules through network module analysis. Here, “module 1” was defined as the most important module and “module 2” as the second most important module if available. Consequently, models using four types of feature datasets were constructed: SCGs, Module 1, Module 2, and Module 1+2. Here, “module 1+2” indicates the union merge of module 1 and module 2.

The samples were divided into a training set (70%) and a test set (30%) to develop the predictive models. five‐fold cross‐validation was employed to enhance model reliability. By iterating over different hyperparameters, the models were evaluated using metrics such as Spearman correlation and AUROC to identify the best model.

In summary, the optimal drug response model was established for each drug by employing 39 regression models, four types of feature datasets, and three feature selection methods.

### Network Proximity of Drug Signatures by Connecting Drug Targets and Gene Signatures

Experimentally validated drug targets were extracted from five comprehensive drug‐target databases: ChEMBL,^[^ ^48^ ^]^ BindingDB,^[^ ^49^ ^]^ GtopDB,^[^ ^50^ ^]^ DrugBank,^[^ ^51^ ^]^ and DGiDB.^[^ ^52^ ^]^ Additionally, 243,603 PPIs involving 16,677 proteins were collected from a manually curated human protein–protein interaction (PPI) network. This interactome incorporated data from various sources, including IntAct,^[^ ^53^ ^]^ InnateDB,^[^ ^54^ ^]^ PINA,^[^ ^55^ ^]^ HPRD,^[^ ^56^ ^]^ BioGRID,^[^ ^57^ ^]^ PhosphositePlus,^[^ ^58^ ^]^ KinomeNetworkX,^[^ ^59^ ^]^ INstruct,^[^ ^60^ ^]^ SignaLink2.0,^[^ ^61^ ^]^ and MINT.^[^ ^62^ ^]^ These databases encompassed a range of PPI data derived from experimental and computational approaches. All interactions were represented as undirected edges in the network.

Let *T* = (*t*_1,_ *t*_2, _…) denote the set of targets for a given drug, and *G* = (*g*_1,_ *g*_2, _…) represent the set of signature genes for that drug. The network proximity between signature genes and drug targets in the PPI network is defined as:

(1) $$d\left(T,G\right)=\frac{1}{\left|\left|T\right|\right|}\sum_{t\in T}min_{g\in G}d\left(t,g\right)$$

where d(t, g) is the shortest path length between target t in T and signature gene g in G. After iterating through all nodes in the signature gene set G, we sum and average the minimum distances, characterizing the interaction between targets and signature genes.

### Deconvolution Analysis to Infer Leukemic Cell Abundances

The EPIC algorithm was used to estimate the fractions of 11 leukemic cell types in pre‐therapy bulk RNA samples. EPIC (version 1.1.5),^[^ ^24^ ^]^ a widely used deconvolution tool, was obtained from GitHub (https://github.com/GfellerLab/EPIC) and was run with default parameters according to the user manual. Although previous studies have demonstrated that EPIC could accurately quantify various immune cells from solid tumor samples,^[^ ^24^ ^]^ a simulation analysis was conducted to evaluate EPIC's performance in enumerating different leukemic cells using our 231‐gene signatures. A total of 2,529 single cells from single‐cell RNA sequencing (scRNA‐seq) data^[^ ^25^ ^]^ were collected as artificial bulk RNA samples for reference. The abundances of the 11 leukemic cell types of 92 patient samples from the BeatAML cohorts was further extrapolated. The EPIC analysis in this study utilized a leukemia‐specific reference matrix curated by Zhang et al.,^[^ ^28^ ^]^ which focused on 11 well‐characterized AML cell types (e.g., HSC‐like, GMP‐like, monocyte‐like) derived from single‐cell studies.

### Prioritize Optimal Drugs According to the Pharmacogenomic Features of Individual Patients

To verify the predictive ability of the models, it was applied to AML patients and thus prioritized optimal drugs in the scRNA‐seq cell. The relevant FASTQ files of AML patient scRNA‐seq was first downloaded from the “Genome Sequence Archive for Human” at the BIG Data Center of the Beijing Institute of Genomics, Chinese Academy of Sciences.^[^ ^28^ ^]^ The downloaded data were preprocessed and analyzed using 10x Genomics’ Cell Ranger software (version 8.0.1), which included quality control, sequence alignment, gene expression quantification, and cell identification. During processing, the default Human GRCh38 reference genome was used for alignment.

Subsequent data processing utilized the Seurat package (version 5.1.0)^[^ ^63^ ^]^ for quality control, normalization, dimensionality reduction, clustering, and doublet removal. First, raw single‐cell data were read to create a Seurat object. Initial quality control excluded low‐quality cells (those with fewer than 300 genes detected, fewer than 1000 transcripts, or mitochondrial gene content exceeding 20%). The data were then normalized using the LogNormalize method, followed by the selection of 2000 highly variable genes. Scaling was applied to all genes to remove potential systemic biases. Principal component analysis (PCA) was performed, and the top 20 principal components were used for dimensionality reduction and visualization (UMAP). DoubletFinder was employed to predict and remove doublet cells. Finally, the Seurat object was streamlined to retain only the gene expression matrix and normalized data, ensuring clean and reliable datasets. The CCAIntegration method was used to integrate different data layers, with integrated results stored in a new dimensionality‐reduced space to address batch effects. The merged RNA data layers were combined with dimensionality reduction results via the JoinLayers method.

The FindAllMarkers function in Seurat was applied to cell type annotation, identifying marker genes for each cell cluster. Selection criteria required that genes be detected in at least 25% of cells within a cluster and exhibit a log2 fold change greater than 0.5 between clusters, with statistical significance assessed using the MAST method. The top 10 marker genes with the highest expression variation in each cluster were selected to annotate cell populations. Malignant cells were distinguished from normal cells using inferCNV,^[^ ^64^ ^]^ which analyzed copy number variation (CNV) patterns to identify malignant cells.

A larger scale of 87 drug models was leveraged to predict drug responses for each cell in the scRNA‐seq data. The area under the curve (AUC) was used as the evaluation metric, with lower AUC values indicating stronger drug efficacy. AUC values were transformed for comparative analysis, and drug response profiles for each cell were visualized.

### Statistical Analysis

Statistical analysis was performed using R (version 4.3.3) and Python (version 3.8.13). Drug‐gene correlations were assessed using Spearman's rank correlation (FDR‐adjusted) via the SciPy package. Regression modeling was implemented with Scikit‐learn using 5‐fold nested cross‐validation. HotNet algorithm to identify gene modules that exhibited close interactions within the PPI network. Cell deconvolution was performed by both the EPIC algorithm (https://github.com/GfellerLab/EPIC) and CIBERSORTx algorithm (https://cibersortx.stanford.edu/). Seurat package (version 5.1.0) in R was utilized for quality control, normalization, dimensionality reduction, clustering, and doublet removal. Malignant cells were distinguished from normal cells using the inferCNV package in R (https://github.com/broadinstitute/infercnv). Kaplan–Meier and log‐rank test analysis are performed by the “survival” and “survmine” packages in R. Group comparisons of employed Mann‐Whitney tests and t‐test, with significance denoted as ^*^ *P* < 0.05, ^**^ *P* < 0.01, ^***^ *P* < 0.001, and ^****^ *P* < 0.0001. The correlation analysis was performed primarily using Pearson correlation, with R indicating the correlation coefficient, and the *P*‐value representing the significance of the correlation as follows: ^*^ *P* < 0.05, ^**^ *P* < 0.01, ^***^ *P* < 0.001, and ^****^ *P* < 0.0001. Our cohort consisted of 520 patients from the OHSU study, encompassing 87 different drugs.

### Code Availability

The code used for the analysis was developed in Python (Version 3.8.13), R (Version 4.3.3), and is available in a GitHub repository (https://github.com/19900321/NetAML).

## Conflict of Interest

The authors declare no conflict of interest.

## Author Contributions

Y.W. and R.L. contributed equally to this work. J.T. performed project administration, supervision, methodology, conceptualization, editing, and funding acquisition. Y.W. contributed to data analysis, methodology, conceptualization, data visualization, writing of original draft, editing, and funding acquisition. R.L. contributed to data analysis, data visualization, writing of original draft, and editing. X.L. contributed to data analysis, visualization, and data collection. N.T. provided review and guidance. Y.Z. was responsible for data analysis and data visualization. C.Y. was responsible for data analysis. S.T. provided review and writing.

## Supporting information



Supporting Information

Supplemental Table 1

Supplemental Table 1

## Data Availability

The data that support the findings of this study are openly available in Github at https://github.com/19900321/NetAML, reference number 0.
