# Supplementary material for: A Network‐Driven Framework for Drug Response Precision Prediction of Acute Myeloid Leukemia
Source: Adv Sci (Weinh). 2025 Jul 11;12(36):e06447. doi: 10.1002/advs.202506447 (PMC12463123; doi:10.1002/advs.202506447)
Supplement: Supplementary file 1 — Supporting Information [file ADVS-12-e06447-s001.docx]

Supporting Information

**A Network-Driven Framework for Drug Response Precision Prediction of Acute Myeloid Leukemia**

*Yinyin Wang^a,b#,*^, Rui Liu^a,#^, Yinnan Zhang^a^, Xiang Luo^a^, Chengzhuang Yu^a^, Shentong Fang^a^, Ninghua Tan^a,*^, Jing Tang^b,*^*

a Department of TCMs Pharmaceuticals, School of Traditional Chinese Pharmacy, China Pharmaceutical University, Nanjing, 211198, PR China

b Research Program in Systems Oncology, Faculty of Medicine, University of Helsinki, FI-00014, Finland

# These authors made equal contributions to this work.

* Corresponding authors

E-mail: [jing.tang@helsinki.fi](mailto:jing.tang@helsinki.fi); [yinyin.wang@cpu.edu.cn](mailto:yinyin.wang@cpu.edu.cn); [nhtan@cpu.edu.cn](mailto:nhtan@cpu.edu.cn);

**Keywords:** Acute Myeloid Leukemia, Drug sensitivity prediction, Precision medicine, Network-based analysis, Machine learning, FLT3 Inhibitor

| **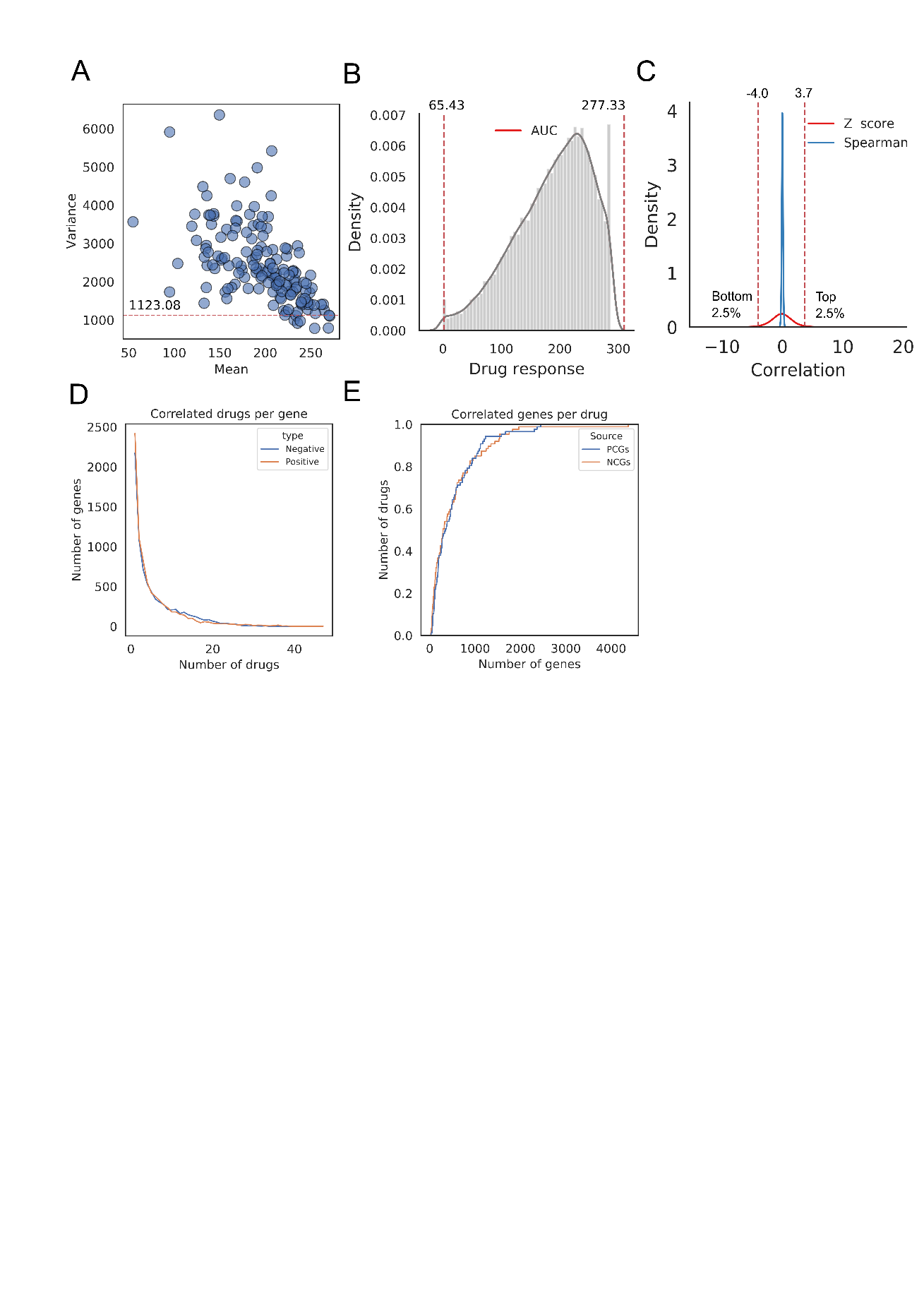** |
| --- |
| **Figure S1.** The statistics on the distribution of drug response and correlated genes. A) Distribution of mean and variance of AUC from 87 drugs from the BeatAML cohort. This line represents the threshold for the bottom 5% with the most minor variance, and its purpose is to help identify regions where the variance among different samples changes minimally. B) Distribution of AUC of 520 patients, with the red line representing the threshold for the top and bottom 0.025% of values at 65.43 and 277.33. C) The density of the correlation coefficient (blue line) and Z-score from the Z-transformed correlation (red line) with the dashed red line at -4.0 and 3.7, indicating the top and bottom 0.025%. D) The density of the number of correlated drugs per gene. E) The density of the number of correlated genes per drug. |

| **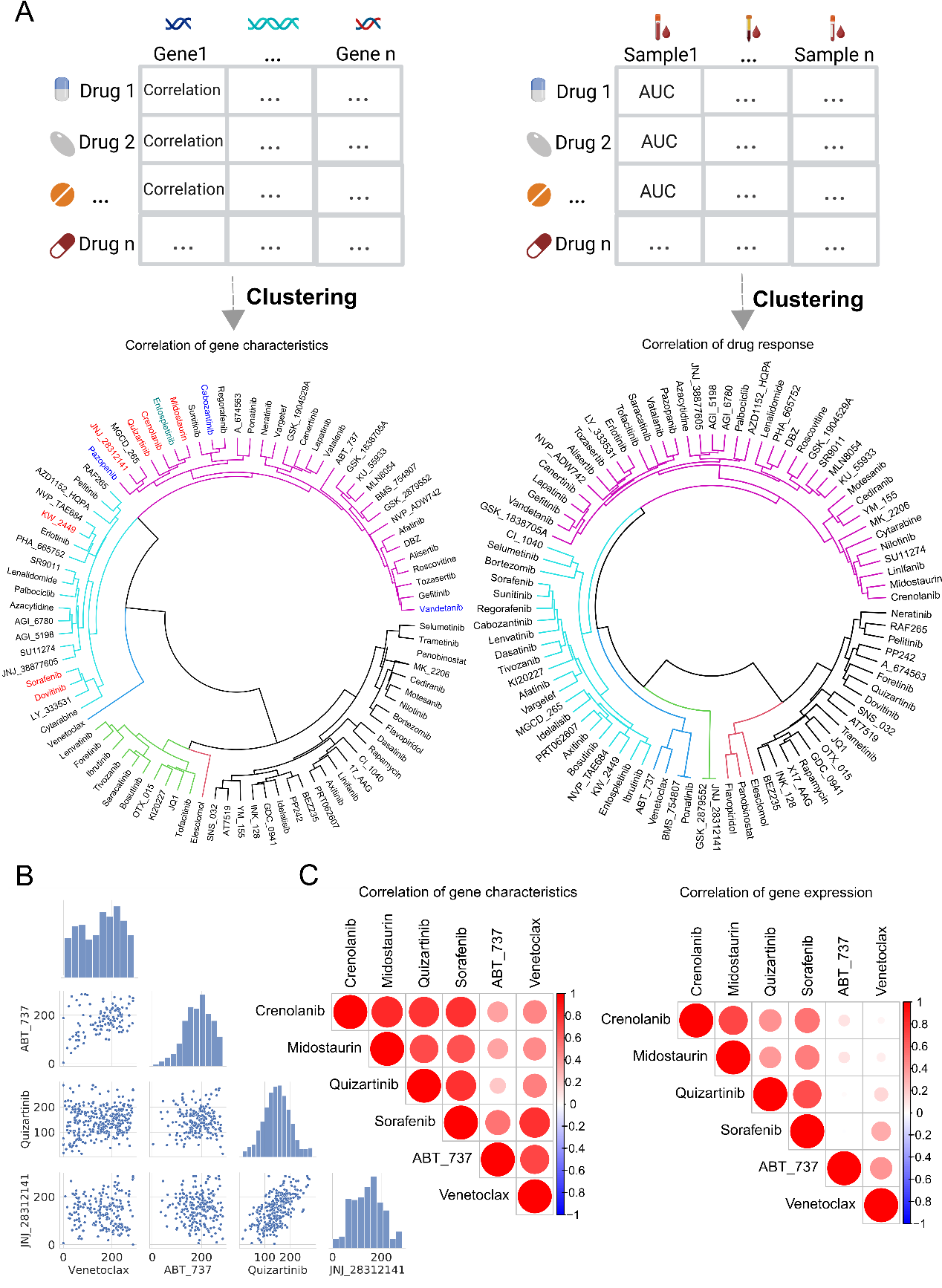** |
| --- |
| **Figure S2.** The correlation analysis between the drug and genes. A) Hierarchical clustering with complete linkage and a Euclidean distance matrix on a panel of gene-drug correlations (Left panel) and drug response correlations (Right panel). The dendrogram was cut to define six drug clusters for further analysis. The correlation of gene characteristics was constructed by clustering based on the correlation between drugs and genes. In contrast, the correlation of drug response was constructed by clustering based on the AUC values between drugs and samples. The drugs highlighted in color are those that were explicitly analyzed in the original manuscript. The drugs marked in red are FLT3 inhibitors, those in blue are VEGFR inhibitors, and those in green are kinase inhibitors. B) Scatter plot of AUC between the FLT3i drugs and Bcl-2i on 520 samples. C) The Pearson correlation on gene characteristic (Left panel) and drug response (Right panel) of FLT3i and BCL-2i drug responses. |

| **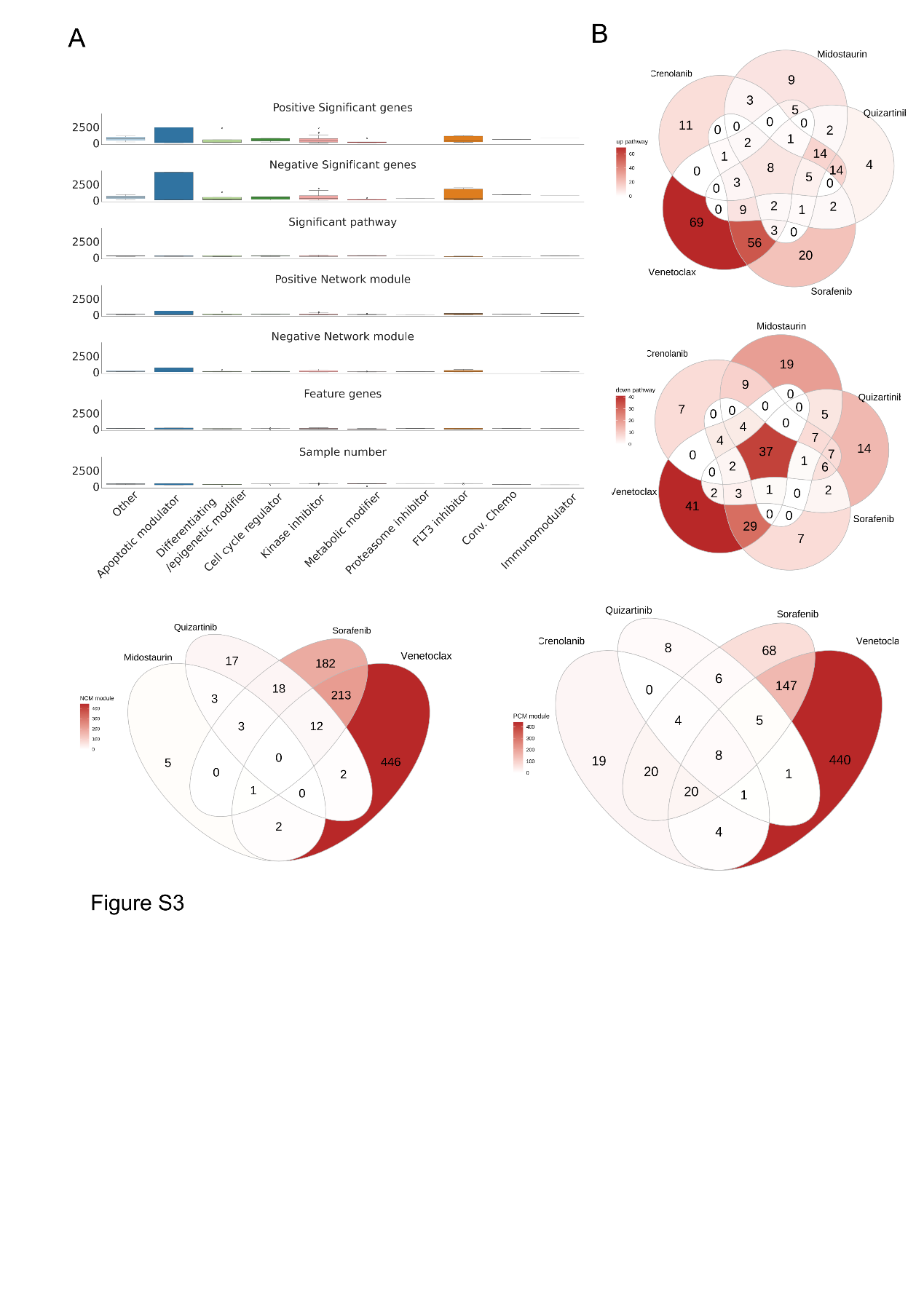** |
| --- |
| **Figure S3.** Statistical analysis of the network module. A) The distribution of PCGs, NCGs, PMGs, NMGs, feature genes, and samples among different classifications of drugs. B) The overlapping situation of up pathways and down pathways of FLT3i Crenolanib, Midostaurin, Quizartinib, and Sorafenib. |

| **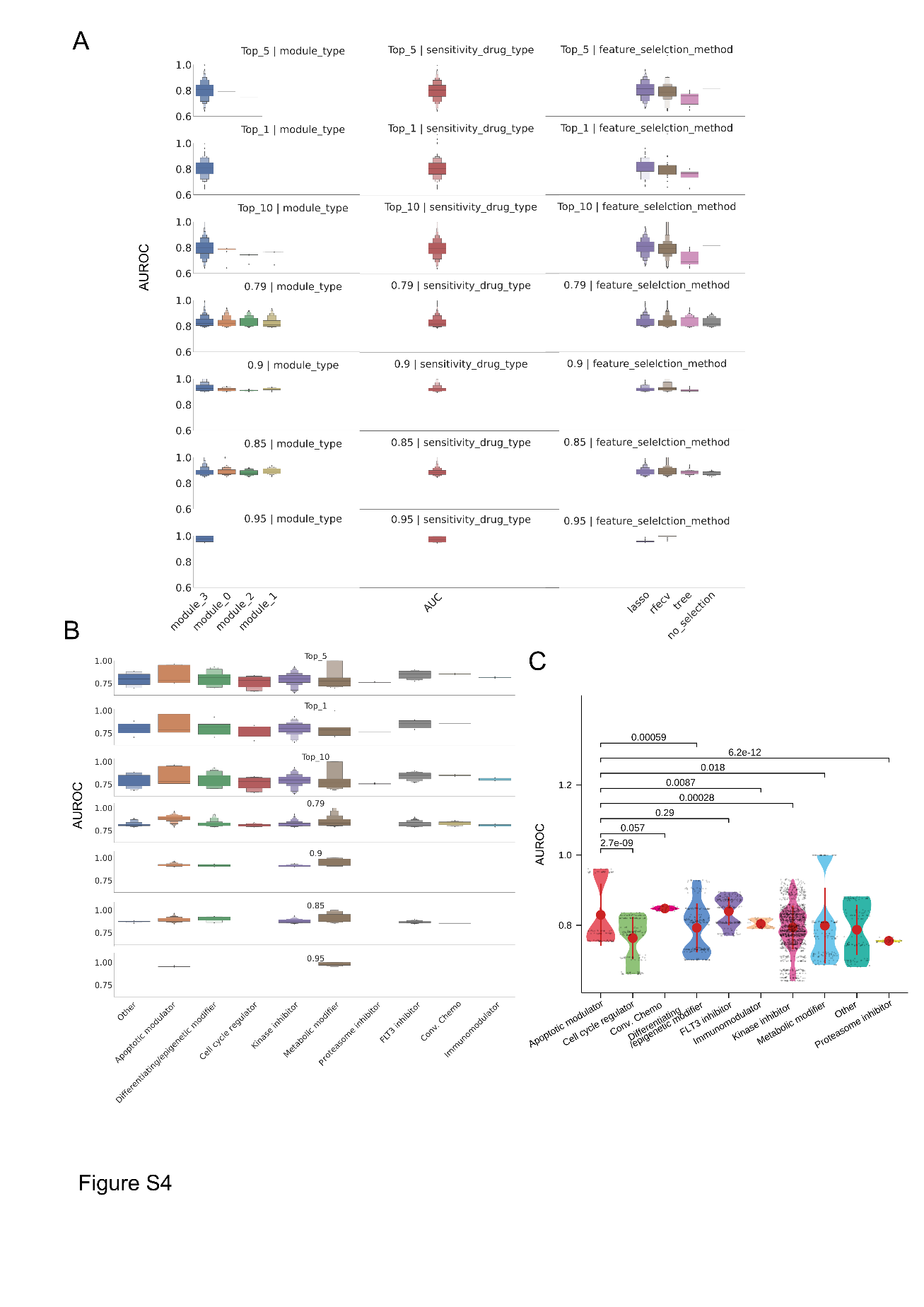** |
| --- |
| **Figure S4.** Model performance comparison. A) The AUROC performance of various hyperparameters in LR models on BeatAML patients, including feature types and feature selection methods. B) The AUROC performance on drugs from different functional classes. C) The AUROC performance of the top 10 models across drugs from different functional classes with the Mann-Whitney test. |

| **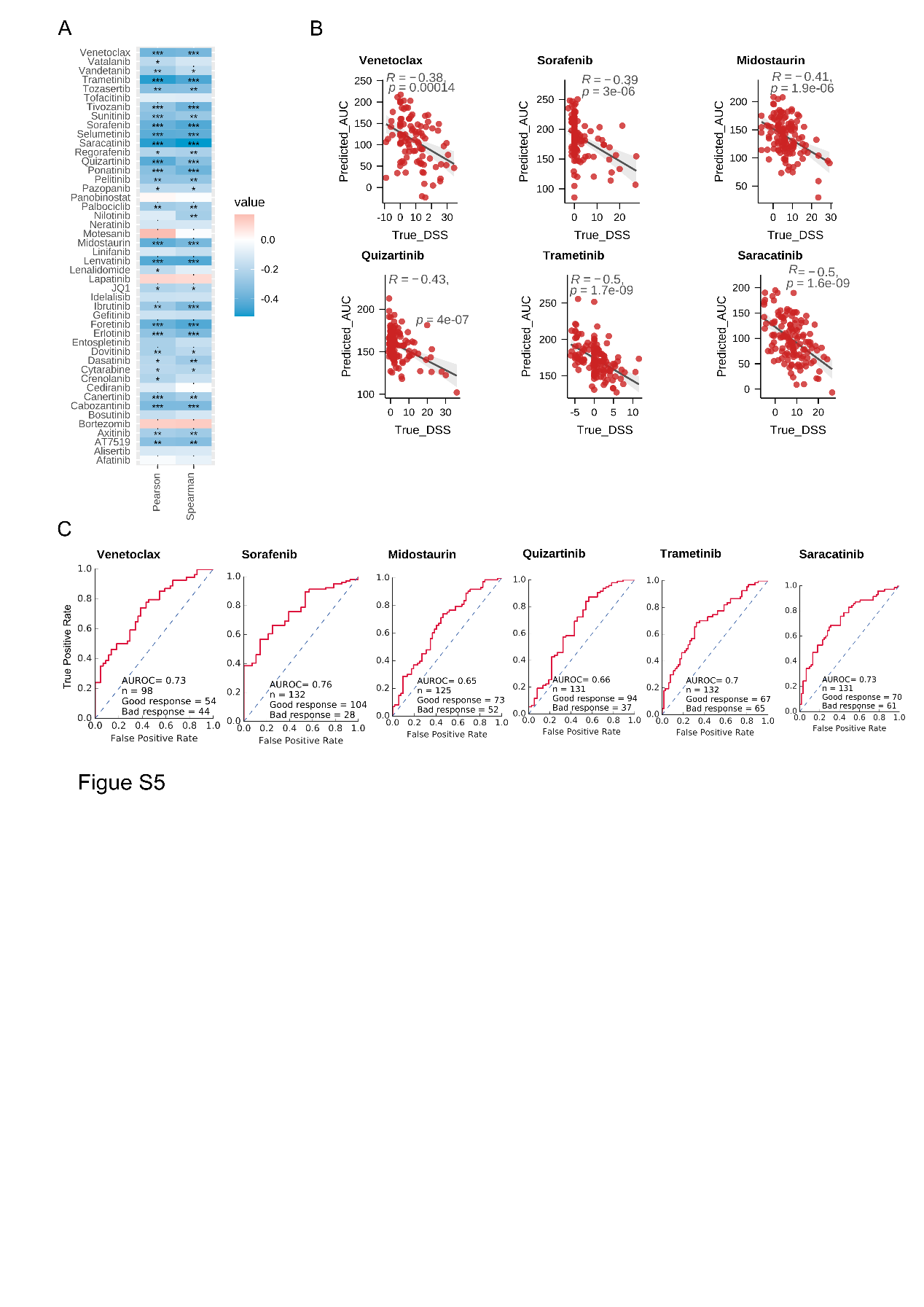** |
| --- |
| **Figure S5.** External validation of the drug sensitivity prediction model of AML patients by the FPMTB cohort. A) The Pearson and Spearman correlation between AUROC and the predicted AUC and DSS score, * *P* < 0.05，** *P* < 0.01, *** *P* < 0.001. B) The Pearson correlation evaluation with predicted AUC as the Y axis and true DSS as the drug response. C) The AUROC evaluation with predicted AUC and observed DSS. |

| **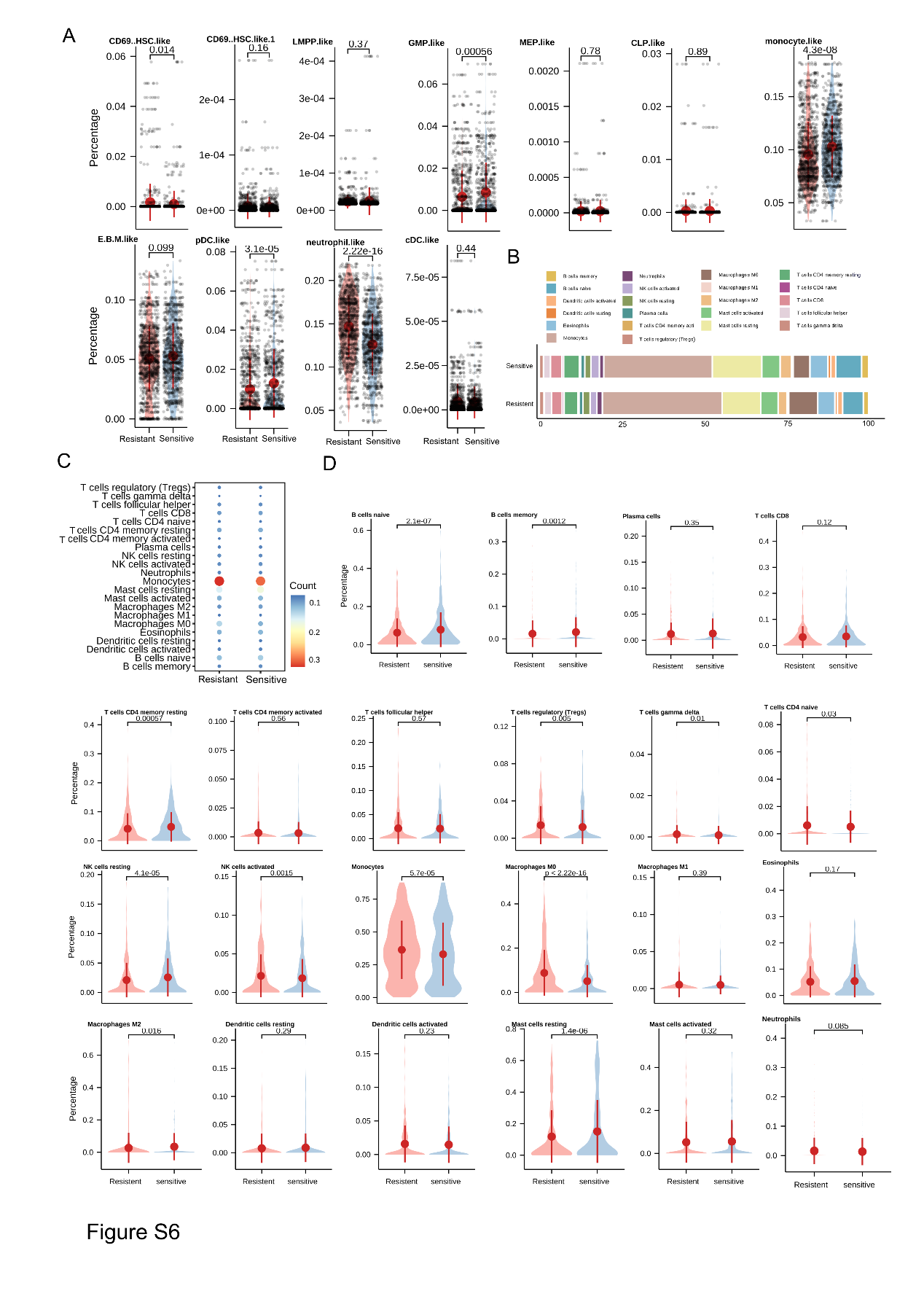** |
| --- |
| **Figure S6.** Comparison of cell heterogeneity among sensitive and resistant patients. A) Comparison of cell type percentages via the EPIC method cell deconvolution among sensitive and resistant patients using t-tests. B) The averaged cell type percentages of sensitive and resistant patients were separately analyzed using the CIBERSORTx method for cell deconvolution. C) The average percentage of cell types from the CIBERSORTx method among sensitive and resistant patients. D) Comparison of cell type percentages from the CIBERSORTx method among sensitive and resistant patients using t-tests. |
| **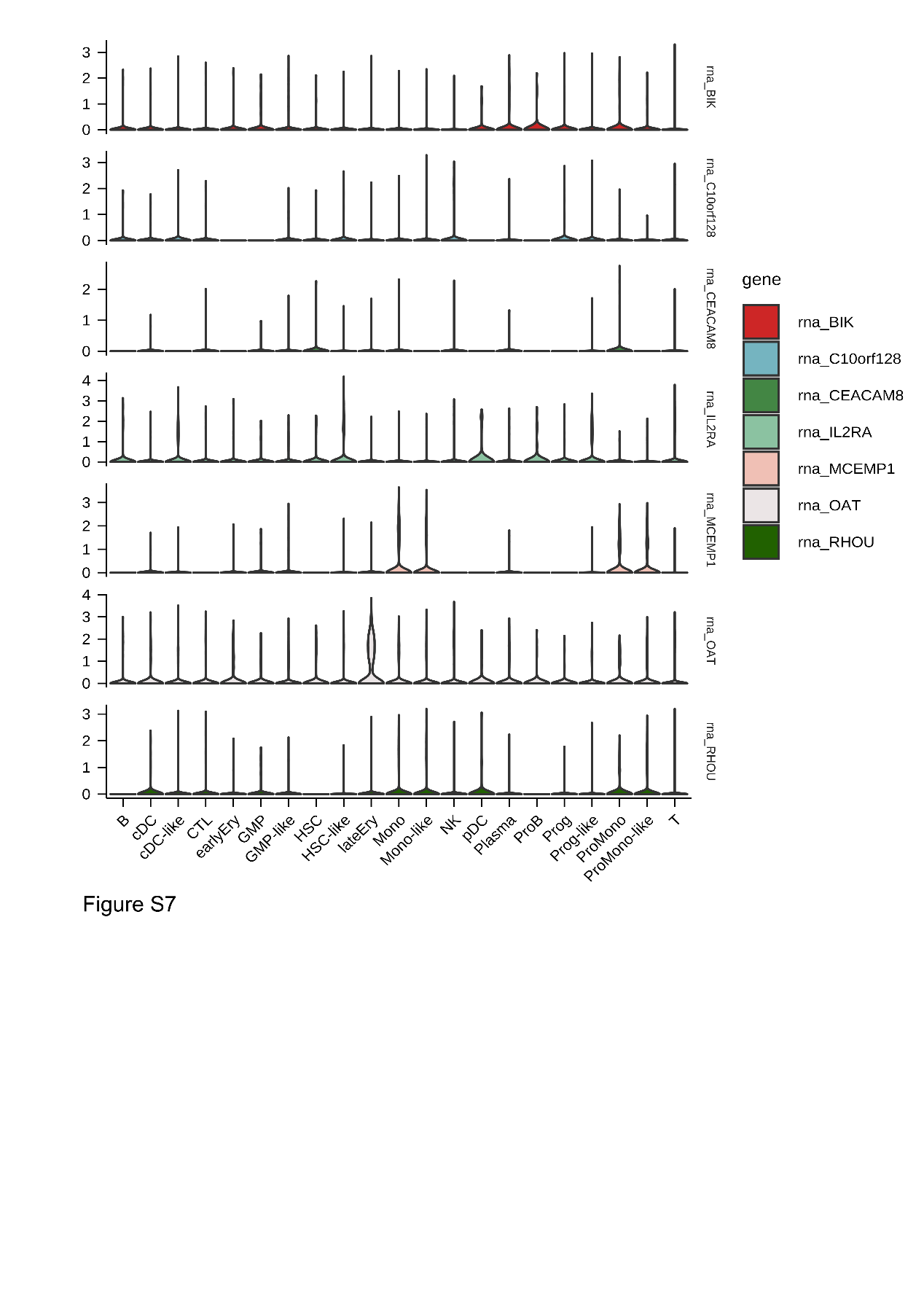** |
| **Figure S7.** Comparison of signature genes for FLT3i across different Cell subtype populations. |

| 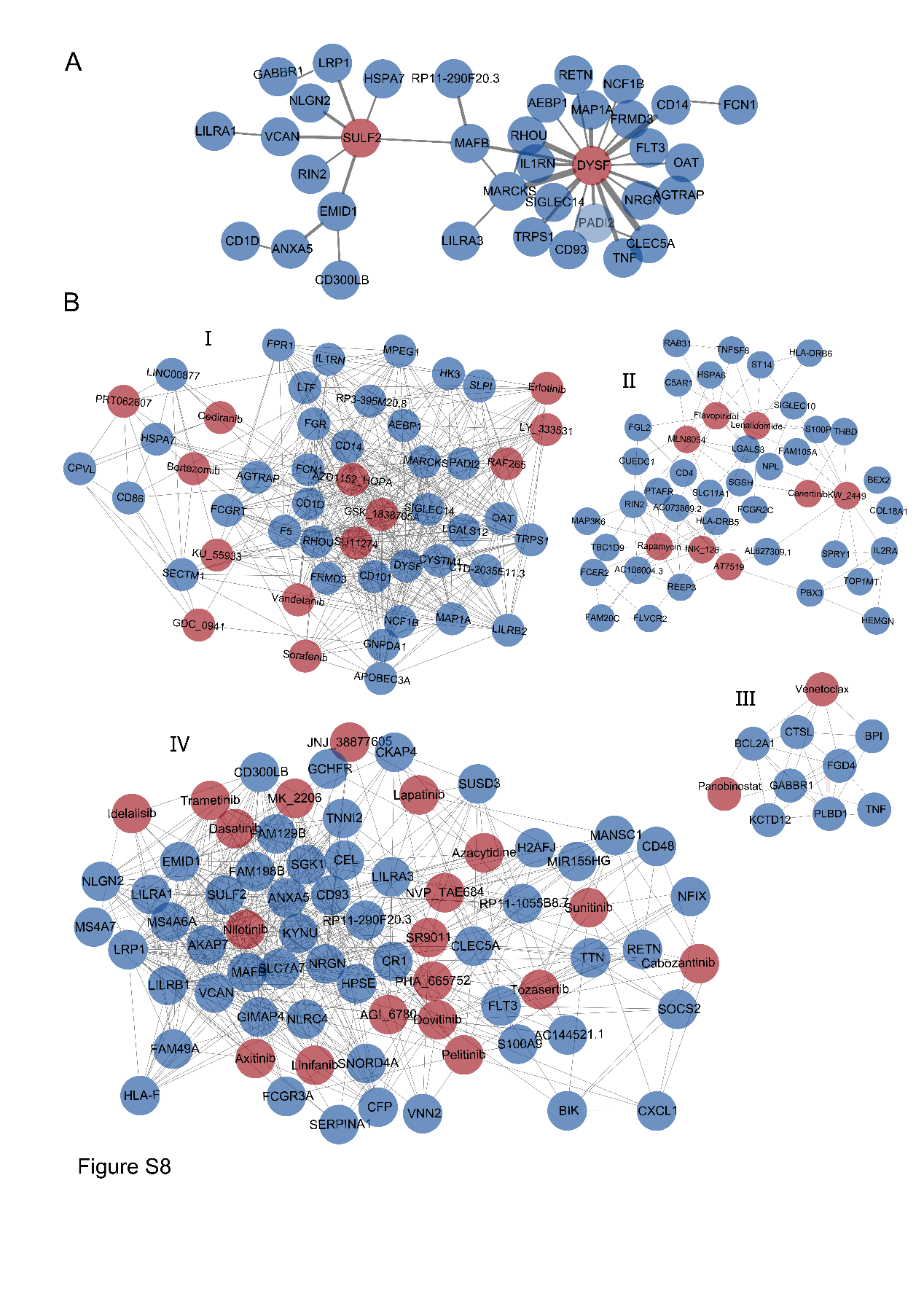 |
| --- |
| **Figure S8.** Core network modules of drug signature genes. A) The gene-gene pairs patterns that consist of genes as signature genes in at least five drugs. B) Three core modules from community analysis on the drug-gene signature network. |

| 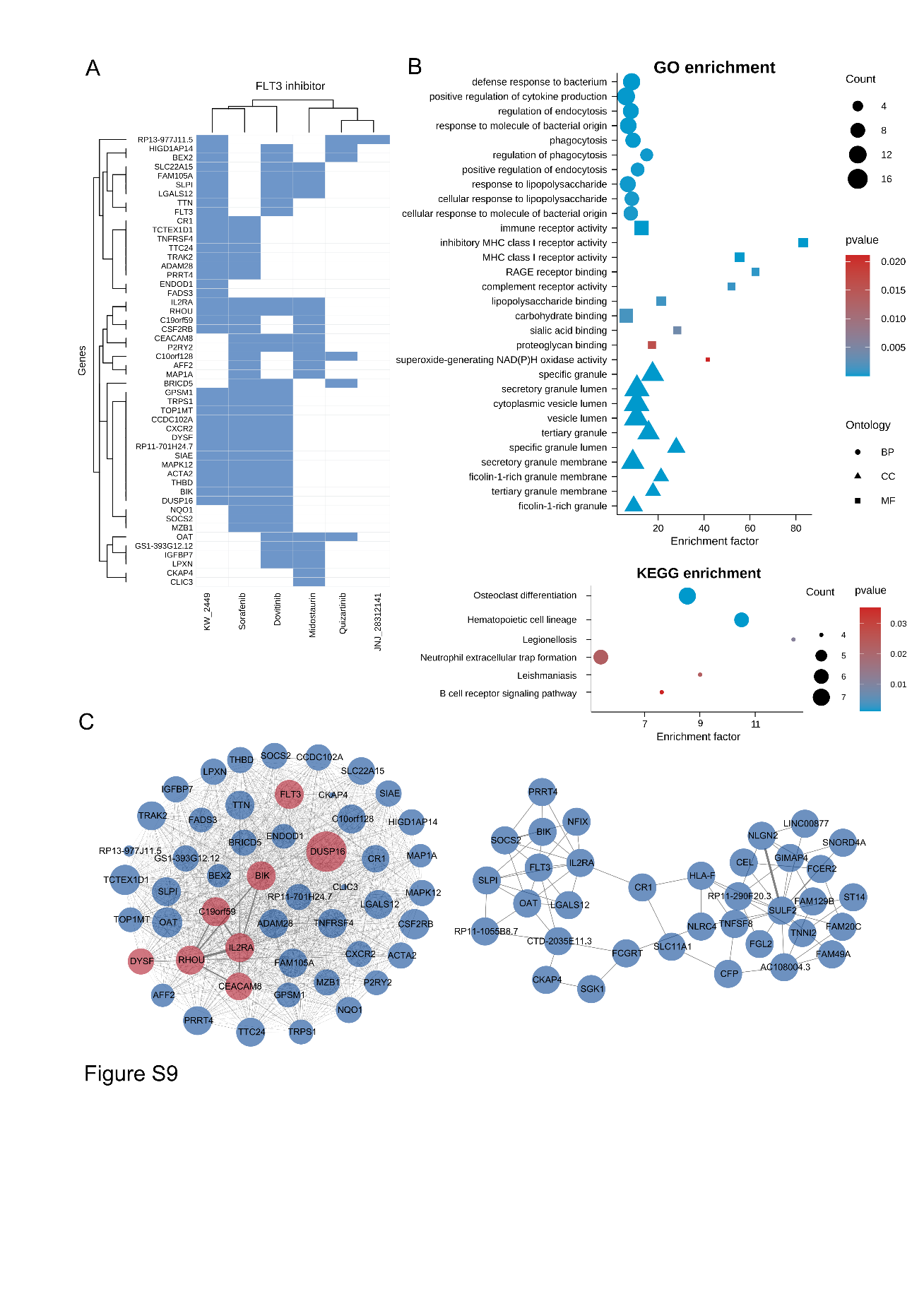 |
| --- |
| **Figure S9.** Modules of FLT3i drug signature genes. A) Heatmap of gene signatures of FLT3i drugs. B) GO terms and KEGG pathways associated with signatures of FLT3i. C) Gene-gene pairs patterns co-exist among the top 10 drugs. |

| 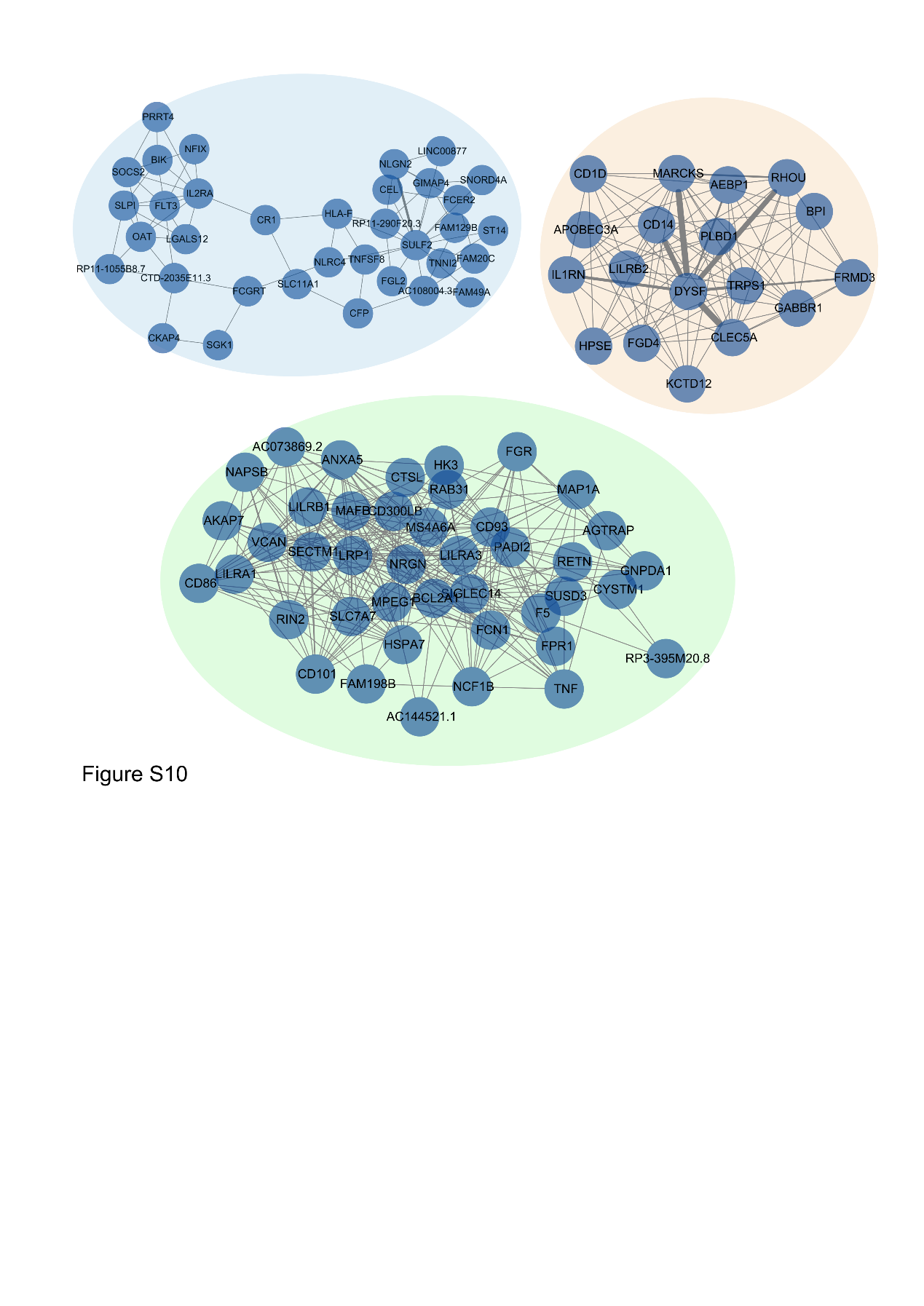 |
| --- |
| **Figure S10.** The core modules from the gene-gene interactions network of signature genes. |

| 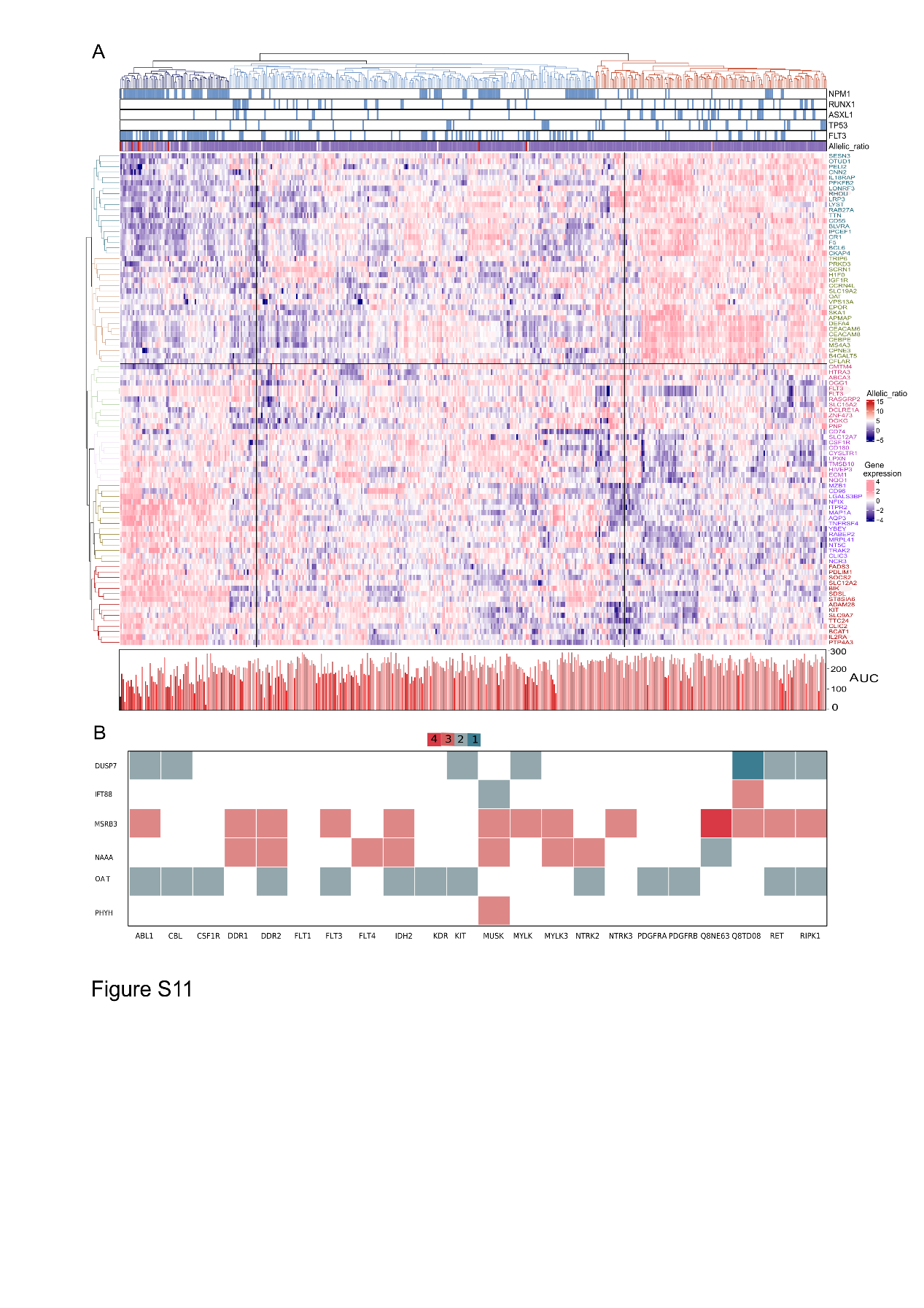 |
| --- |
| **Figure S11.** Network analysis on target genes and feature genes of Quizartinib among PPI networks. A) The gene expression heatmap of the target genes and feature genes of Quizartinib-treated patients. B) The distance matrix between target genes and feature genes of Quizartinib. |

| 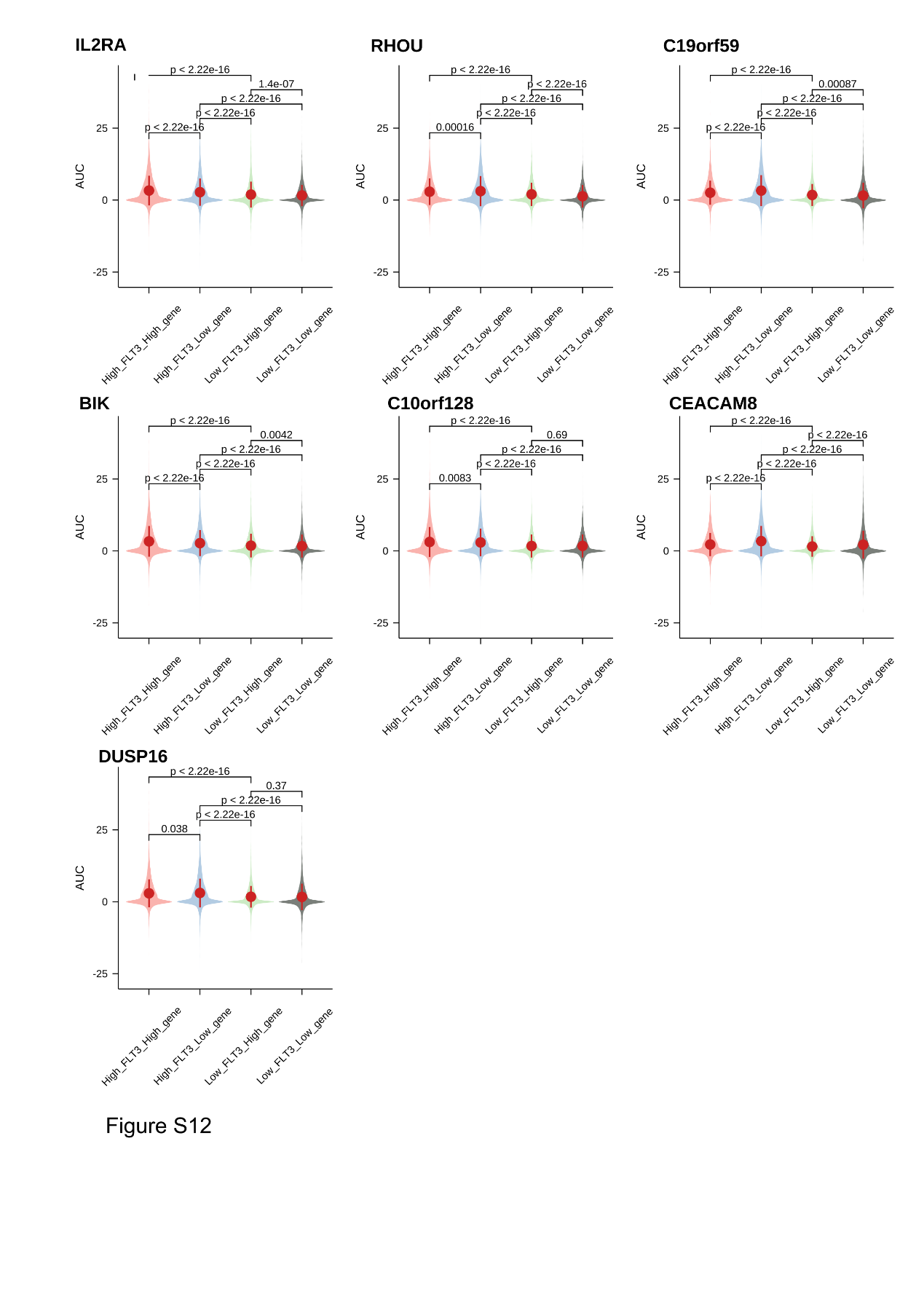 |
| --- |
| **Figure S12.** The comparison of drug response DSS of FPMTB patients between different groups of FLT3 gene signatures with a t-test. |

| 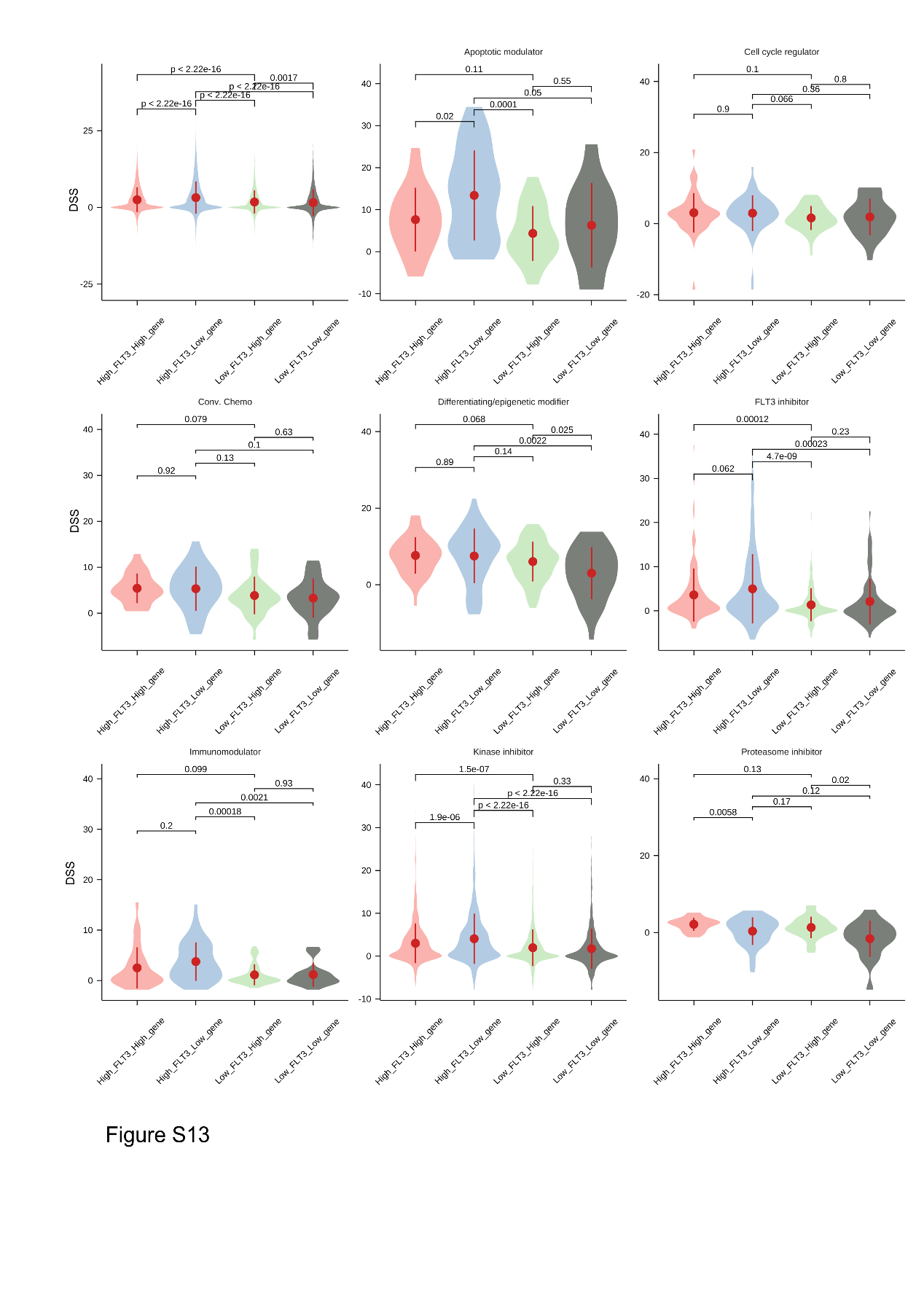 |
| --- |
| **Figure S13.** Comparison of drug sensitivity between different types of drugs by grouping FLT3-mutated patients from the FPMTB cohort by FLT3 and C19ORF59 genes with a t-test |

| 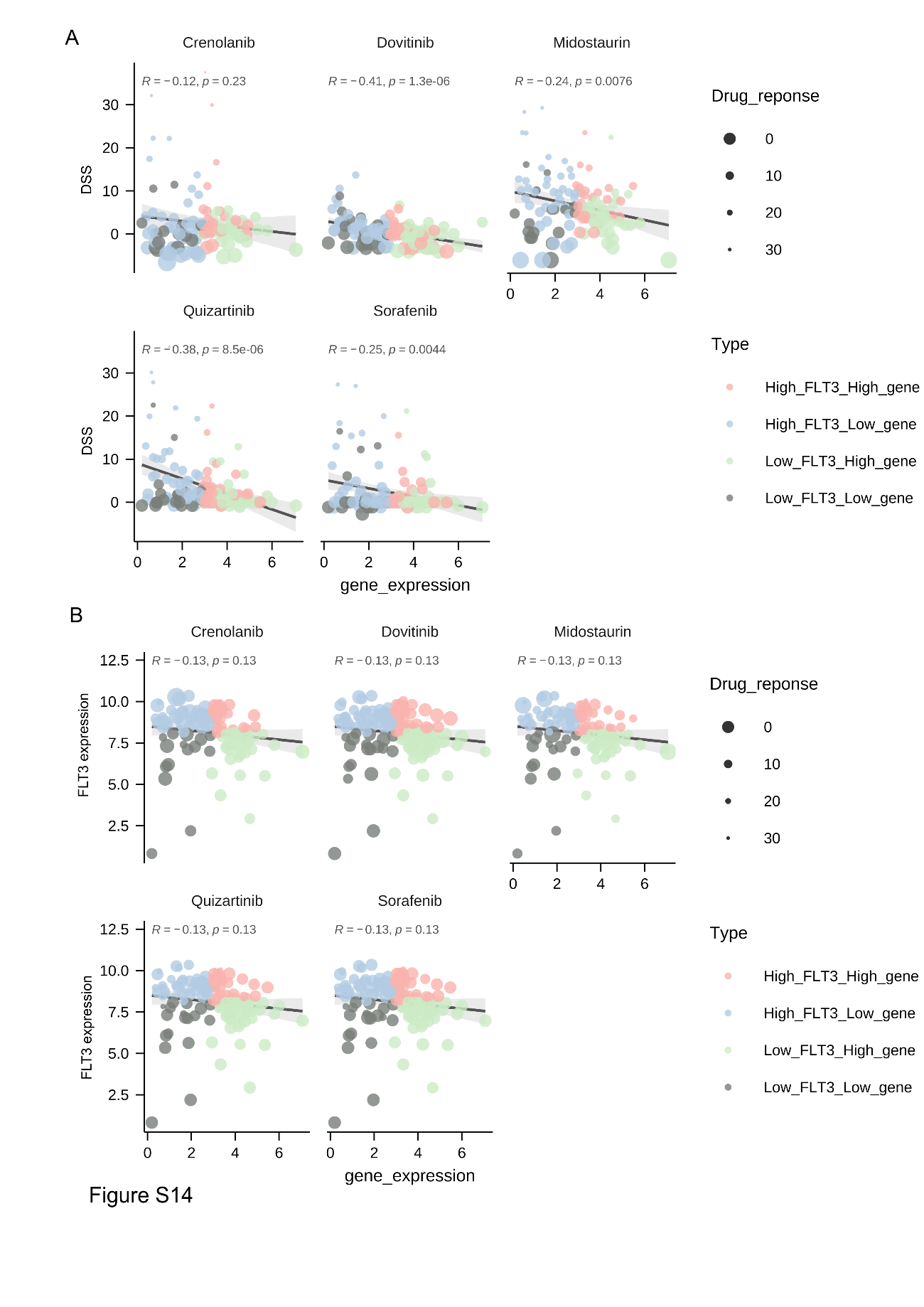 |
| --- |
| **Figure S14.** C19ORF59 expression is associated with the drug response of FLT3i in the FPMTB cohort. A) The Pearson correlation between the expression of C19ORF59 and seven FLT3i drugs, with color indicating different groups of genes and size representing the drug response. B) The Pearson correlation between the expression of C19ORF59 and seven FLT3 genes, with color indicating different groups of genes and size representing the drug response. |

| 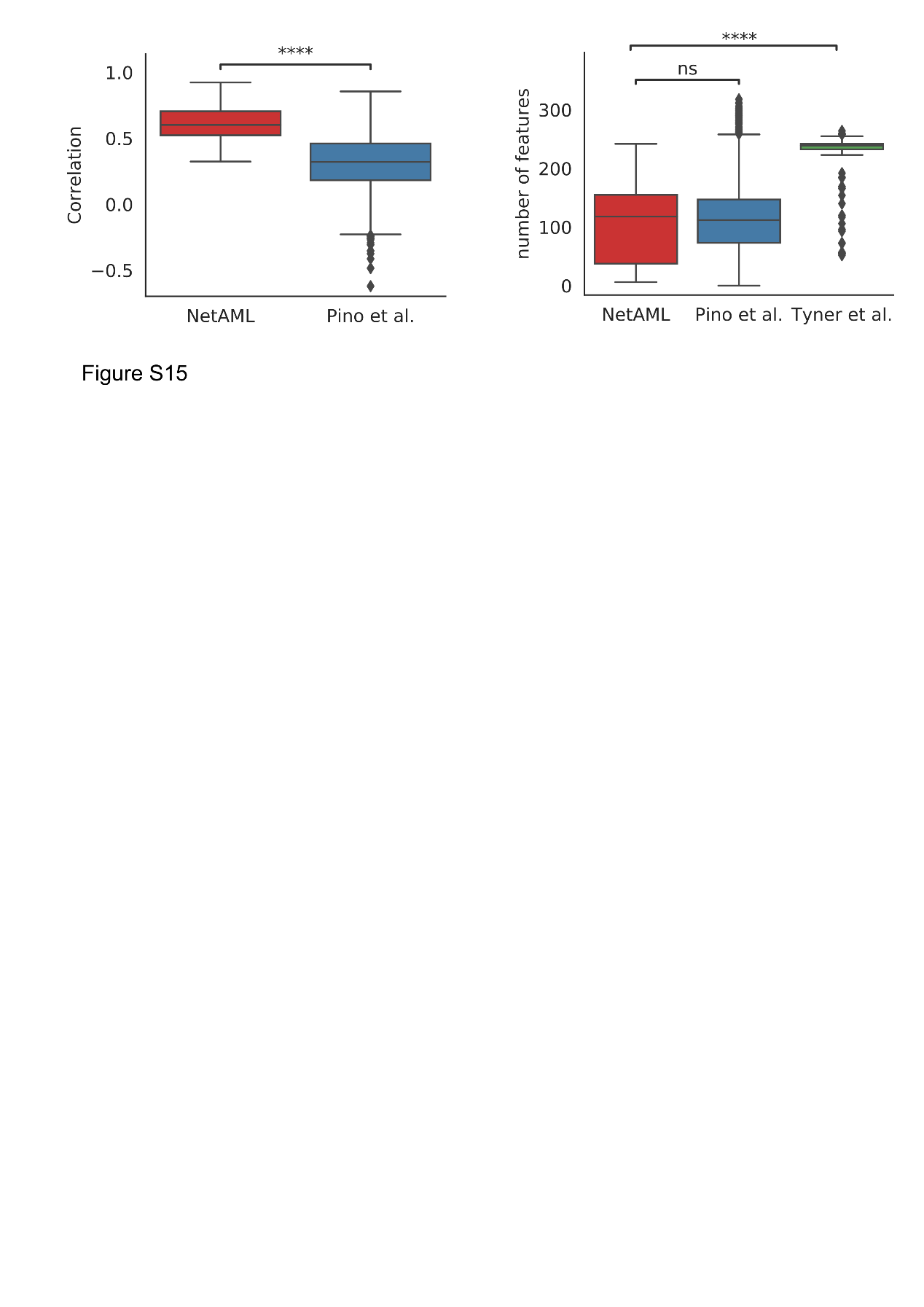 |
| --- |
| **Figure S15.** Performance comparison of NetAML with the Benchmarking model from Pino et al and Tyner et al. Group comparisons employed two-sided Mann-Whitney U tests, with significance denoted as ns > 0.05, **P* < 0.05, ***P* < 0.01, ****P* < 0.001, and *****P* < 0.0001. |

| Supplementary Table S1. The hyperparameter of 39 regression models for model training. | |
| --- | --- |
| Model name | hyperparameter |
| RANSACRegressor | min_samples=0.5, residual_threshold=None, max_trials=100 |
| RidgeCV | alphas=(0.1, 1.0, 10.0), cv=None |
| LinearSVR | epsilon=0.0, C=1.0, loss='epsilon_insensitive' |
| GradientBoostingRegressor | n_estimators=100, learning_rate=0.1, max_depth=3, loss='squared_error' |
| SGDRegressor | loss='squared_error', penalty='l2', alpha=0.0001 |
| HuberRegressor | epsilon=1.35, alpha=0.0001 |
| PoissonRegressor | alpha=1.0, max_iter=100 |
| Ridge | alpha=1.0 |
| TransformedTargetRegressor | default |
| LinearRegression | default |
| Lars | n_nonzero_coefs=500, fit_intercept=True |
| NuSVR | nu=0.5, C=1.0, kernel='rbf' |
| Lasso | alpha=1.0 |
| TweedieRegressor | power=0.0，alpha=1.0 |
| SVR | C=1.0, kernel='rbf', epsilon=0.1 |
| LassoLarsIC | criterion='aic', normalize='deprecated' |
| BayesianRidge | alpha_1=1e-6, alpha_2=1e-6, lambda_1=1e-6, lambda_2=1e-6 |
| ElasticNetCV | l1_ratio=0.5, alphas=(0.1, 1.0, 10.0) |
| GammaRegressor | alpha=1.0, max_iter=100 |
| ElasticNet | alpha=1.0, l1_ratio=0.5 |
| LassoCV | alphas=None, cv=None |
| LassoLarsCV | max_iter=500, cv=None |
| LassoLars | alpha=1.0, max_iter=500 |
| LarsCV | cv=None |
| DummyRegressor | strategy='mean' |
| OrthogonalMatchingPursuitCV | cv=None,n_nonzero_coefs=None |
| OrthogonalMatchingPursuit | n_nonzero_coefs=None, tol=None |
| KernelRidge | alpha=1, kernel='linear' |
| GaussianProcessRegressor | kernel=RBF(), alpha=1e-10 |
| AdaBoostRegressor | n_estimators=50, learning_rate=1.0, loss='linear' |
| ExtraTreesRegressor | n_estimators=100, max_depth=None, min_samples_split=2 |
| RandomForestRegressor | n_estimators=100, max_depth=None, min_samples_split=2 |
| MLPRegressor | hidden_layer_sizes=(100, ), activation='relu', solver='adam' |
| PassiveAggressiveRegressor | C=1.0, loss='epsilon_insensitive', max_iter=1000 |
| LGBMRegressor | n_estimators=100, learning_rate=0.1, max_depth=-1 |
| BaggingRegressor | n_estimators=10, base_estimator=None |
| HistGradientBoostingRegressor | max_iter=100, learning_rate=0.1, max_depth=None |
| KNeighborsRegressor | n_neighbors=5, weights='uniform', algorithm='auto' |
| XGBRegressor | n_estimators=100, learning_rate=0.3, max_depth=6, objective='reg:squarederror' |
